# Genome-wide associations for benign prostatic hyperplasia reveal a genetic correlation with serum levels of PSA

**DOI:** 10.1038/s41467-018-06920-9

**Published:** 2018-11-08

**Authors:** Julius Gudmundsson, Jon K. Sigurdsson, Lilja Stefansdottir, Bjarni A. Agnarsson, Helgi J. Isaksson, Olafur A. Stefansson, Sigurjon A. Gudjonsson, Daniel F. Gudbjartsson, Gisli Masson, Michael L. Frigge, Simon N. Stacey, Patrick Sulem, Gisli H. Halldorsson, Vinicius Tragante, Hilma Holm, Gudmundur I. Eyjolfsson, Olof Sigurdardottir, Isleifur Olafsson, Thorvaldur Jonsson, Eirikur Jonsson, Rosa B. Barkardottir, Rafn Hilmarsson, Folkert W. Asselbergs, Gudmundur Geirsson, Unnur Thorsteinsdottir, Thorunn Rafnar, Gudmar Thorleifsson, Kari Stefansson

**Affiliations:** 1 deCODE genetics/AMGEN, 101 Reykjavik, Iceland; 2 Landspitali-University Hospital, 101 Reykjavik, Iceland; 3 Faculty of Medicine, University of Iceland, 101 Reykjavik, Iceland; 4 School of Engineering and Natural Sciences, University of Iceland, 101 Reykjavik, Iceland; 5 Department of Cardiology, Division Heart & Lungs, University Medical Center Utrecht, University of Utrecht, 3584 CX Utrecht, The Netherlands; 6 The Clinical Laboratory in Mjodd, 109 Reykjavik, Iceland; 7 Department of Clinical Biochemistry, Akureyri Hospital, 600 Akureyri, Iceland; 8 Laboratory of Cell Biology, Department of Pathology, Landspitali University Hospital, 101 Reykjavik, Iceland; 9 Biomedical Centre, Faculty of Medicine, University of Iceland, 101 Reykjavik, Iceland; 10 Durrer Center for Cardiovascular Research, Netherlands Heart Institute, 3511 EP Utrecht, The Netherlands; 11 Institute of Cardiovascular Science, Faculty of Population Health Sciences, University College London, London, WC1E 6BT UK; 12 Farr Institute of Health Informatics Research and Institute of Health Informatics, University College London, London, NW1 2DA UK

## Abstract

Benign prostatic hyperplasia and associated lower urinary tract symptoms (BPH/LUTS) are common conditions affecting the majority of elderly males. Here we report the results of a genome-wide association study of symptomatic BPH/LUTS in 20,621 patients and 280,541 controls of European ancestry, from Iceland and the UK. We discovered 23 genome-wide significant variants, located at 14 loci. There is little or no overlap between the BPH/LUTS variants and published prostate cancer risk variants. However, 15 of the variants reported here also associate with serum levels of prostate specific antigen (PSA) (at a Bonferroni corrected *P* < 0.0022). Furthermore, there is a strong genetic correlation, *r*_g_ = 0.77 (*P* = 2.6 × 10^−11^), between PSA and BPH/LUTS, and one standard deviation increase in a polygenic risk score (PRS) for BPH/LUTS increases PSA levels by 12.9% (*P* = 1.6×10^−55^). These results shed a light on the genetic background of BPH/LUTS and its substantial influence on PSA levels.

## Abstract (web summary)

Elderly males are often affected by benign prostatic hyperplasia and associated lower urinary tract symptoms (BPH/LUTS), but their link to prostate cancer risk is not well defined. Here, a genome-wide association study of BPH/LUTS patients from Iceland and the UK found 23 significant variants at 14 loci, and 15 of these variants associate with prostate specific antigen, which is linked to prostate cancer risk.

## Introduction

Benign prostatic hyperplasia (BPH), the nonmalignant enlargement of the prostate, and associated lower urinary tract symptoms (LUTS) are common medical conditions among elderly males. Autopsy studies have unveiled a histological prevalence of the disease of: 8, 50, and 80%, in the fourth, sixth, and ninth decades of life, respectively^1^. BPH contributes to bladder outlet obstruction, leading not only to bothersome LUTS but can, if untreated, be detrimental to patients’ health by affecting bladder and kidney function. Furthermore, BPH/LUTS is associated with depression, and diminished health-related quality of life; based on sleep, psychological condition, activities in daily life, and sexual activities^2–4^. The high prevalence of BPH/LUTS and its effect on various other health related conditions results in a high annual health-care cost, both for patients and societies. This cost is likely to rise dramatically over the next few decades as life expectancy is on the rise in most countries. The detailed molecular pathogenesis of BPH/LUTS has not been well established. However, in addition to age, inflammation^5^, sex hormones^6^, and metabolic factors^7^ have all been implicated. Furthermore, genetic variation is a strong risk factor for developing BPH/LUTS. A study of men who underwent surgery for BPH younger than 64 years of age, reported that other male relatives and brothers of probands had a four- and six fold increase, respectively, of age-specific risks of BPH surgery^8^. In addition, twin studies report the concordance rate ratios for BPH/LUTS to range between 2.2 and 6.9 depending on the specificity of symptom definition^9, 10^. Despite this relatively strong genetic component of the disease, only very few suggestively associated sequence variants have been reported for BPH/LUTS^11–14^.

In order to search for variants conferring risk of symptomatic BPH/LUTS, we performed a genome-wide association study (GWAS) in two study groups, coming from Iceland and the UK Biobank^15^. We report here genome-wide significant results for 23 genetic variants, located at 14 loci, conferring risk of symptomatic BPH/LUTS.

## Results

### GWAS analysis

The GWAS of the Icelandic BPH/LUTS dataset included 9443 men with symptomatic BPH/LUTS and 104,000 controls. Men with symptomatic BPH/LUTS were defined as individuals undergoing transurethral resection of the prostate (TURP), as well as men older than 50 years, repeatedly using drugs for treating BPH/LUTS belonging to the G04C group of the Anatomical Therapeutic Chemical (ATC) classification (for example: tamsulosin, finasteride, and dutasteride).

The UK Biobank dataset consists of 11,178 men with BPH/LUTS according to hospital-based diagnosis, as well as 176,541 controls not known to have been diagnosed with BPH/LUTS. For a description of the genotyping and imputation of the Icelandic and UK Biobank samples (see the Methods section).

Per-allele odds ratios (ORs) and two-sided *P*-values for all ~42.9 million variants in the GWASs of both study groups were obtained using a logistic regression model. We then conducted a fixed-effect meta-analysis including the Icelandic and the UK results with 20,621 patients and 280,541 controls, in total.

### Association with BPH/LUTS

**Fig. 1:**
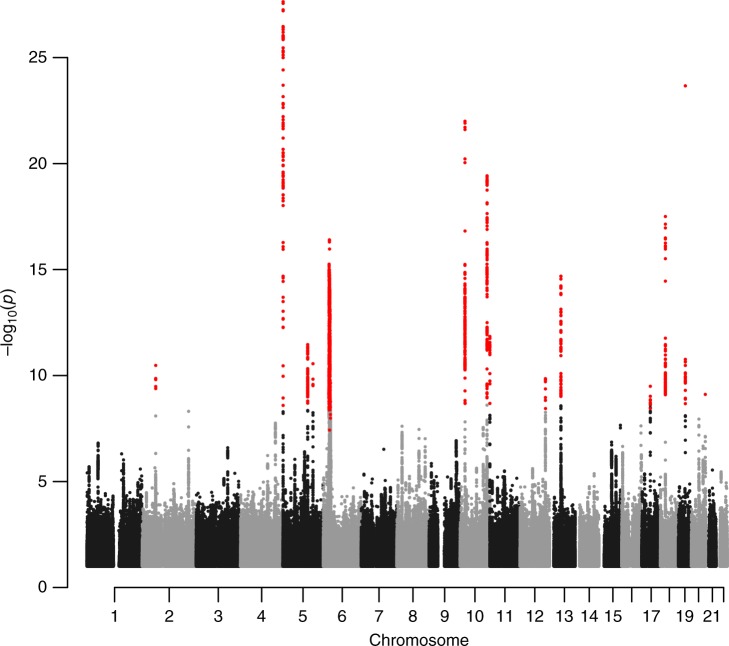
A Manhattan plot of the combined BPH/LUTS GWAS results. The Manhattan plot shows variants with two-sided *P*-value < 0.10 (obtained using a logistic regression model) and high imputation information score (info > 0.90) from the BPH/LUTS meta-analysis of GWAS data from 20,621 patients and 280,541 controls of European ancestry, coming from Iceland and the UK. Shown are negative log_10_-transformed two-sided *P*-values from the unconditional analysis (*y*-axis) over 22 autosomes (*x*-axis). Dots colored in red denote variants that surpass our genome-wide significance thresholds (ranging between 1.9 × 10^−7^ and 5.9 × 10^−10^), defined using a weighted Bonferroni procedure based on functional impact of classes of variants

An initial screening of the GWAS results revealed 14 variants, at 14 loci, surpassing our genome-wide significance criteria (Table 1, Supplementary Tables 1 and 2, Fig. 1, Supplementary Fig. 1 and 2). The threshold for genome-wide significance in the present study was corrected for multiple testing using a weighted Bonferroni procedure based on functional impact of classes of variants^16^ (for our GWAS the significance thresholds range between 1.9 × 10^−7^ and 5.9 × 10^−10^ depending on functional annotations; see Methods). For all 14 variants the effect estimates in the Icelandic and UK samples were highly consistent and no significant heterogeneity was detected when considering the number of variants tested (a Bonferroni corrected *P*-value of 0.05/14 = 0.0036).

**Table 1:** Results from the meta-analysis of Icelandic and UK GWAS of symptomatic BPH/LUTS and from the the conditional analysis for loci with multiple variants

Locus	Marker (EA/OA)	Covariate	Annotation/nearby gene(s)	EAF	*P*_het_/*I*^2^(%)	Meta-analysis results	
						OR (95% c.i.)	*P*-value
2p16.1	rs2556378 (T/G)	rs10180282	Intron variant/*BCL11A*	0.154	0.37/0	1.12 (1.08, 1.15)	3.4 × 10^−12^
2p16.1	rs10180282^a^ (T/C)	rs2556378	Intergenic variant/*BCL11A*	0.456	0.58/0	1.06 (1.03, 1.08)	8.7 × 10^−7^
5p15.33	rs381949 (A/G)	rs2853677	Intron variant/*CLPTM1L*	0.415	0.86/0	0.90 (0.88, 0.92)	4.9 × 10^−19^
5p15.33	rs2853677^a^ (G/A)	rs381949	Intron variant/*TERT*	0.421	0.44/0	1.09 (1.06, 1.11)	1.7 × 10^−12^
5q22.1	rs10054105 (G/T)	na	Intergenic variant/*STARD4*	0.213	0.65/0	0.91 (0.88, 0.93)	3.5 × 10^−12^
5q31.1	rs677394 (G/C)	na	Intron variant/*C5orf66*, *H2AFY*	0.123	0.034/78	0.88 (0.85, 0.92)	2.9 × 10^−11^
6p22.1	rs200476 (T/A)	na	Intergenic variant/*HIST1H2BL*	0.162	0.23/30	0.88 (0.85, 0.90)	3.9 × 10^−17^
10p12.31	rs148678804 (A/G)	rs7906649	Intergenic variant/*DNAJC1*	0.035	0.17/48	1.27 (1.19, 1.35)	3.0 × 10^−14^
10p12.31	rs7906649^a^ (G/A)	rs148678804	Intergenic variant/*EBLN1*	0.286	0.71/0	1.07 (1.04, 1.10)	2.1 × 10^−7^
10q26.12	rs11199879 (C/T)	rs4548546 and rs2981575	Intergenic variant/*FGFR2*	0.252	0.021/81	1.14 (1.11, 1.17)	5.7 × 10^−23^
10q26.12	rs4548546^a^ (T/C)	rs11199879 and rs2981575	Intron variant/*WDR11*	0.310	0.20/40	1.11 (1.08, 1.13)	2.0 × 10^−16^
10q26.12	rs2981575^a^ (G/A)	rs11199879 and rs4548546	Intron variant/*FGFR2*	0.427	0.97/0	0.94 (0.92, 0.96)	6.0 × 10^−8^
11p15.5	rs72878024 (A/G)	na	Missense variant/*ODF3*	0.080	0.20/40	0.85 (0.82, 0.89)	1.4 × 10^−12^
12q24.21	rs2555019 (T/C)	rs8853	Intergenic variant/*TBX5*	0.456	0.82/0	0.93 (0.91, 0.95)	2.4 × 10^−11^
12q24.21	rs8853^a^ (C/T)	rs2555019	3-prime UTR variant/*TBX3*	0.494	0.75/0	1.07 (1.05, 1.10)	1.4 × 10^−9^
13q14.3	rs1638703 (C/G)	rs6561599	Intron variant/*DLEU1*	0.256	0.57/0	1.10 (1.07, 1.13)	1.1 × 10^−13^
13q14.3	rs6561599^a^ (C/G)	rs1638703	Upstream gene variant/*RNASEH2B*	0.371	1.0/0	0.94 (0.92, 0.96)	1.8 × 10^−7^
17q12	rs11651052 (A/G)	na	Intron variant/*HNF1B*	0.470	0.24/29	0.93 (0.91, 0.95)	3.2 × 10^−10^
18q11.2	rs9958656 (T/C)	rs17670370	Intergenic variant/*GATA6*	0.430	1.0/0	1.11 (1.08, 1.13)	4.3 × 10^−19^
18q11.2	rs17670370^a^ (G/T)	rs9958656	Intergenic variant/CTAGE1	0.262	0.24/28	1.07 (1.04, 1.10)	1.6 × 10^−7^
19q12	rs11084596 (C/T)	na	Intergenic variant/*THEG5*	0.356	0.34/0	0.88 (0.86, 0.90)	2.1 × 10^−24^
20q13.33	rs200383755 (C/G)	rs6061244	Missense variant/*GATA5*	0.0091	0.53/0	0.67 (0.59, 0.77)	3.2 × 10^−9^
20q13.33	rs6061244^a^ (C/G)	rs200383755	Intron variant/*GATA5*	0.386	0.16/49	0.94 (0.92, 0.96)	5.7 × 10^−8^

Shown is the effect allele (EA), the other allele (OA), the simple average effect allele population frequency (EAF), the allelic odds ratio (OR) for the effect allele with upper and lower 95% confidence intervals (c.i.) and the two-sided *P*-value for association testing between variants and disease, which was performed using the likelihood ratio statistic. Results from the two study groups were combined using a Mantel-Haenszel model (see Methods). Annotation is according to Variant Effect Predictor (VEP). Shown are also the *P*-value for the heterogeneity (*P*_het_) between the two study groups and the heterogeneity statistic (*I*^2^) representing the fraction of variability due to heterogeneity between study groups. rs200383755 had an imputation information score of 0.99 and 0.88 in the Icelandic and UK datasets, respectively. All other markers listed had imputation information score >0.95. Results for markers pertaining to loci with more than one association signal are shown after conditioning on a relevant covariate. Markers at loci with no additional association signal do not have any applicable covariate (na) and the results are the unconditioned association result from the GWAS of symptomatic BPH/LUTS

^a^Markers discovered in the conditional analysis

In order to search for additional association signals, we performed a stepwise CGTA-COJO^17^ conditional analysis at the 14 newly discovered risk loci. We found 9 secondary association signals at 8 loci after conditioning on the lead marker at each of the 14 loci. For the variants identified with the COJO method, we then performed a conditional analysis using individual genotypes (Table 1, Supplementary Table 2, and Methods). The significance threshold for the combined results from the conditional analyses was set at *P* < 1 × 10^−6^ since, when performing the conditional analyses we tested approx. 50,000 markers (Bonferroni correction: 0.05/50,000 = 1.0 × 10^−6^). For information about pairwise linkage disequilibrium (LD) between lead variants at loci with multiple association signals, see Supplementary Table 3. In total, the unconditional GWAS and conditional analysis returned 23 variants, associated with symptomatic BPH/LUTS in our study. Thereof, 3 are rare or low frequency (with an average minor allele frequency (MAF) ≤ 8%) and 2 of those are missense variants (Table 1). All variants reported in Table 1 had imputation information score > 0.95, except rs200383755, which had an imputation information score of 0.99 and 0.88 in the Icelandic and UK datasets, respectively.

### Bioinformatics and quantitative trait locus analyses of risk variants

Our bioinformatics and expression quantitative trait locus analyses yielded several interesting findings for the newly discovered BPH/LUTS variants. Especially noticeable was the high fraction of risk loci (14 out of 23) with variants identified within regions marked by acetylation of histone H3 at lysine residue K27 (H3K27ac) in prostate epithelial cells. The H3K27ac mark is a well-known marker of active regulatory regions found within enhancers and gene promoters. Below we summarize findings for 3 of the 14 BPH/LUTS risk loci, for a more detailed information about all 23 variants located at the 14 BPH/LUTS risk loci, see Supplementary Note 1, Supplementary Table 4, and Supplementary Data 1 and 2.

**Fig. 2:**
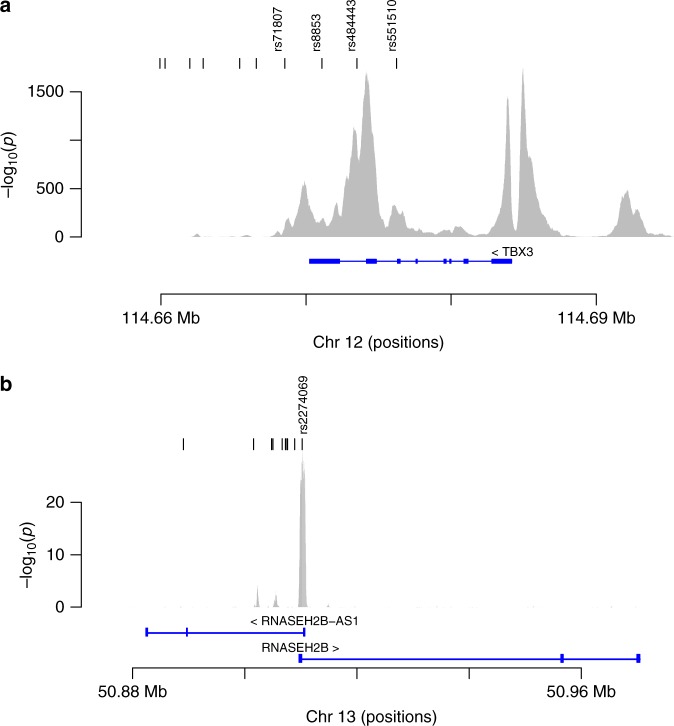
GWAS variants intersecting with regulatory regions defined on the basis of acetylation of histone H3 at lysine residue K27 (H3K27ac). Shown are results for two of the loci reported to associate with BPH/LUTS from an analysis of non-coding risk variants intersecting with regulatory regions defined on the basis of acetylation of histone H3 at lysine residue K27 (H3K27ac), indicative of regulatory regions, in primary prostate epithelial cells. The *y*-axis shows the ChIP-seq signal for the H3K27ac mark represented as negative log_10_ of the *P*-value and the *x*-axis shows the genomic location (hg38). The black tick marks (top of panels **a** and **b**) indicate the position of variants found in strong LD (*r*^2^ > 0.8) with the lead variant, defining an LD class, wherein rs numbers are shown for those residing within H3K27ac significant regions. **a** At 12q24.21 four variants reside within an H3K27ac marked region (rs71807, rs8853, rs484443, and rs551510). **b** At 13q14.3, only one variant, rs2274069, belonging to the LD class of the lead variant resides within a H3K27ac marked region. This is the promoter region for *RNASEH2B*, located within 500 bp from the transcription start site of the gene

The 12q24.21 locus has two independently associated BPH/LUTS variants. rs2555019 is located intergenic and downstream of *TBX5*, a member of a gene family that encodes transcription factors involved in regulation of embryonic developmental processes. The other variant, rs8853, is correlated (*r*^2^ = 0.64) with rs11067228 reported to associate with serum levels of prostate-specific antigen (PSA)^18^ and it is located in the 3′-untranslated region (UTR) of *TBX3*, belonging to the same gene family as *TBX5*. Germline mutations in *TBX3* underlie ulnar mammary syndrome, a rare pleiotropic developmental disorder characterized by altered: upper limbs, apocrine and mammary glands, and genitals^19^. According to the Genotype-Tissue Expression (GTEx) analysis, based on multiple tissues, the expression of *TBX3* is reported to rank second and third highest in bladder and prostate tissues, respectively. Based on our focused analysis of promoters/enhancer regions in prostate epithelial cells we found the 12q24.12 locus (with rs8853 as a lead variant) to intersect with a super-enhancer and to have a clear tissue-specificity with respect to the H3K27ac mark in prostate-derived cells (Fig. 2a). Furthermore, based on a recently developed enhancer-gene target resource, referred to as the Joint Effect of Multiple Enhancers (JEME), *TBX3* is the only candidate target gene, in primary prostate tissue samples, linked to this enhancer element.

rs1638703 and rs6561599 on 13q14.3 are independently associated with BPH/LUTS according to our results. rs1638703 is fully correlated (*r*^2^ = 1) with rs202346, which has been reported to associate with serum levels of PSA^18^ and it is located intronic within the non-protein coding gene *DLEU1*, whereas rs6561599 is located some 5 kb upstream of *RNASEH2B*. The protein encoded by this gene is the non-catalytic B-subunit of RNase H2 endonuclease complex, which is thought to play a role in nucleic acid metabolism to preserve genome stability and to prevent immune activation^20^. Our focused analysis (with rs6561599 as a lead variant) of promoters/enhancers revealed a tissue-specific promoter region for *RNASEH2B*, wherein the H3K27ac mark was particularly prevalent in prostate-derived cells (Fig. 2b).

The 20q13.33 locus also contains two variants independently associated with BPH/LUTS. One of these variants, rs200383755_C, is a missense variant (p.Ser19Trp) in the *GATA5* gene. In our combined study group this variant has a minor allelic frequency of 0.9%, and confers strong protection against BPH/LUTS, with an OR = 0.67 and *P* = 3.2 × 10^−9^ (Table 1). Correspondingly, Icelandic carriers of this variant underwent a TURP treatment 2.7 years older (*P* = 0.013) than non-carriers (see Supplementary Table 5). The *GATA5* gene encodes a transcription factor that contains two GATA-type zinc fingers and is required during cardiovascular development^21^. According to the GTEx Portal, *GATA5* has the highest expression in bladder but its expression is also relatively high in prostate tissue, ranking seventh from the top. The other independently associated variant at 20q13.33 is rs6061244_C (OR = 0.94 and *P* = 5.7 × 10^−8^; Table 1), located intronic in *GATA5*, and as it has no strongly correlated variants (*r*^2^ > 0.75) it can be considered a probable causative variant.

Prostate cancer and BPH/LUTS can coexist in elderly men, e.g. in the Icelandic BPH/LUTS study group 15% of the men have also been diagnosed with prostate cancer and 8.8% in the UK sample set. Two of the BPH/LUTS variants discovered in our study have previously been reported to associate with risk of prostate cancer, i.e. rs2555019 (it has *r*^2^ = 0.81 with rs1270884^22^) located downstream of *TBX5* on 12q24.21, and rs11651052 (which has *r*^2^ = 0.91 with rs4430796^23^) located intronic in *HNF1B* on 17q12. Therefore, we performed a GWAS of BPH/LUTS, where we excluded all men known to have prostate cancer, included in our study groups (see Supplementary Table 6). The results from this analysis did not yield any new genome-wide significant BPH/LUTS loci but the results for rs11651052 on chromosome 17q12 fell well below our threshold of genome-wide significance. The combined unconditional association results for rs11651052_A and the total list of BPH/LUTS (i.e. including men also with prostate cancer) were: OR = 0.93 and *P* = 3.2 × 10^−10^, whereas the unconditional results for men only known to have BPH/LUTS were: OR = 0.95 and *P* = 7.5 × 10^−6^ (see Supplementary Tables 2 and 6). It is therefore possible that our initial BPH/LUTS association signal for rs11651052 was inflated due to a confounding effect from men diagnosed with both BPH/LUTS and prostate cancer (i.e. the association effects for BPH/LUTS and prostate cancer are in the same direction, see Supplementary Table 7). The observed difference could also be due to a chance based on who were and who were not removed from the study group for the purpose of this focused analysis. However, disentangling the BPH/LUTS effect from the prostate cancer effect is likely to be challenging and probably requires a very large sample set, preferably including several populations.

The BPH/LUTS association results for the variant on 12q24.21 (rs2555019_T) became more significant after excluding men diagnosed with both BPH/LUTS and prostate cancer. The unconditioned combined association results for rs2555019_T and the total list of BPH/LUTS (i.e. including men also with prostate cancer) were: OR = 0.93 and *P* = 1.4 × 10^−10^, whereas the unconditioned combined results for men only known to have BPH/LUTS were: OR = 0.92 and *P* = 3.0 × 10^−12^ (see Supplementary Tables 2 and 6). This is probably because the effect estimates for BPH/LUTS and prostate cancer are in the opposite direction (see Supplementary Table 7). However, for clarity and consistency of the data, the results reported in Table 1 for rs2555019 are based on the total list of men with BPH/LUTS (i.e. including men with prostate cancer), same as for the rest of the data in Table 1.

### Genetic correlation between serum levels of PSA and BPH/LUTS

Nine of the BPH/LUTS variants discovered in our study have previously been reported^18, 24^ to be genome-wide significantly associated with serum levels of PSA (Supplementary Tables 7 and 8). These results and the fact that BPH is known^25^ to increase serum PSA levels, prompted us to check the association of all 23 BPH/LUTS variants with serum levels of PSA in a sample set of 33,572 Icelandic males, not known to have been diagnosed with prostate cancer or symptomatic BPH/LUTS. Our analysis showed that in total, 15 of the 23 BPH/LUTS variants reported here also associate with PSA levels at a Bonferroni corrected significance threshold (*P* < 0.0022; see Supplementary Table 7). Moreover, the effect estimates for BPH/LUTS and PSA levels are directionally consistent for all 15 variants (see Supplementary Fig. 3).

We estimated the genetic correlation between serum levels of PSA and BPH/LUTS, using cross-trait LD score regression^26^ and the summary statistics from our GWAS of PSA in Iceland and the corresponding data from the GWAS of BPH/LUTS in the UK samples. Our results show a very strong genetic correlation (*r*_g_ = 0.77; *P* = 2.6 × 10^−11^, see Supplementary Table 9) between PSA levels and BPH/LUTS, across these two study populations. For comparison purposes, we checked the genetic correlation between serum levels of PSA and prostate cancer across the same two study populations. Our results demonstrate a strong genetic correlation (*r*_g_ = 0.41; *P* = 6.1 × 10^−5^) between serum levels of PSA and prostate cancer but still it is much weaker than for BPH/LUTS and PSA levels. For comparison, our results indicate that the genetic correlation (*r*_g_) between BPH/LUTS and prostate cancer is 0.17, although nonsignificant (*P* = 0.18, see Supplementary Table 9).

### Polygenic risk scores

We also calculated polygenic risk scores (PRSs) to estimate the contribution of variants that associate with BPH/LUTS or prostate cancer, respectively, to variation in PSA levels. We used effect estimates from the GWAS of BPH/LUTS and prostate cancer in the UK samples to generate PRSs to correlate with serum levels of PSA in the 18,929 Icelandic men (see Methods). The PRSs for BPH/LUTS and prostate cancer correlate very significantly with PSA levels; each standard deviation (SD) increase in the PRSs corresponds to 12.9% (*P* = 6.0 × 10^−45^) and 16.3% (*P* = 9.8 × 10^−68^) increase in PSA levels, respectively (Table 2a). The effects of the PRSs for BPH/LUTS and prostate cancer on PSA levels are largely independent since, in a joint analysis the effect of both remained highly significant, i.e. 8.6% increase (*P* = 3.0 × 10^−20^) and 13.3% increase (*P* = 4.1 × 10^−43^), respectively (Table 2b). This is consistent with the observation that the PRS for BPH/LUTHS has little predictive power for prostate cancer, and vice versa; one SD increase in the PRS for BPH/LUTS increased the risk for prostate cancer by about 4% (*P* = 0.059), and the same increase of the prostate cancer PRS results in about 5% increase of BPH/LUTS risk (*P* = 0.0027; see Supplementary Table 10). These results demonstrate that variants conferring risk of BPH/LUTS and their effects on PSA levels warrant being taken into consideration when interpreting measurements of individual PSA levels, performed in order to screen for prostate cancer.

**Table 2:** Results from testing the association between polygenic risk scores based on UK data, and a phenotype status, based on Icelandic data

PRSs	Phenotype	Effect (*β*)	*P*-value	PSA_increase/PRS_SD (%)	95% c.i. (%)
**(a) Separately**					
PC	PSA levels	0.089	9.8 × 10^−68^	16.3	(14.3, 18.3)
BPH/LUTS	PSA levels	0.071	6.0 × 10^−45^	12.9	(10.9, 14.8)
**(b) Jointly**					
PC	PSA levels	0.074	4.1 × 10^−43^	13.3	(11.3, 15.3)
BPH/LUTS	PSA levels	0.049	3.0 × 10^−20^	8.6	(6.7, 10.5)

Shown are results from testing the association of polygenic genetic risk scores (PRSs), based on effect estimates from the UK for: prostate cancer (PC) and benign prostatic hyperplasia/lower urinary tract symptoms (BPH/LUTS), for correlation with serum levels of PSA (PSA levels) in 18,929 Icelandic males. Shown are the effect estimates (*β*), the two-sided *P*-values calculated using logistic regression in R (v3.5), the percentage increase in PSA levels for each standard deviation (SD) increase in the PRSs, and the 95% confidence intervals (c.i.)

In section **a** the results are shown separately for the PRSs of prostate cancer (PC) and BPH/LUTS, whereas in section **b** the results are shown jointly (i.e. after being conditioned for each other)

## Discussion

In summary, through a GWAS we have discovered the first set of BPH/LUTS risk variants that surpass a genome-wide significance threshold. The majority (15 out of 23) of the variants reported here also associate with serum levels of PSA. We show that genetic correlation between BPH/LUTS and PSA levels is of a similar magnitude to the genetic correlation between prostate cancer and PSA levels. This underlines the complexity of interpreting the commonly applied PSA test, intended to screen for prostate cancer. Interestingly, the BPH/LUTS variants reported here are largely independent of the previously reported prostate cancer risk variants, highlighting the difference in the etiologies of these two prostate diseases. The drugs currently prescribed for patients with BPH/LUTS do not cure the disease but provide a relief of the symptoms, though that relief does not come without side effects. In order to improve treatment, a better understanding of the basic disease-causing factors is needed. Our results provide several potential focus points for future research within this field.

## Methods

### Study populations

The Icelandic BPH/LUTS study population consists of 9443 men with symptomatic BPH/LUTS and 104,000 controls. Men with symptomatic BPH/LUTS were defined as individuals diagnosed after undergoing TURP between 1983 and 2017 (70% of the total list). Also, included are men older than 50 years repeatedly using drugs in the G04C group of the ATC classification (for example, tamsulosin, finasteride, and dutasteride) for treating BPH/LUTS between the years 2003 and 2009 (30% of the total list). The BPH/LUTS patients had a mean age of 71 years based on age at first TURP treatment or youngest age in prescribed drug list. Controls were males not known to have symptomatic BPH/LUTS. The Icelandic prostate cancer GWAS group consisted of 5897 men diagnosed with prostate cancer (mean age at diagnosis is 71 years) according to a nationwide list from the Icelandic Cancer Registry (ICR) and the controls were 102,276 males absent from the same list. The Icelandic study group used for GWAS of serum levels of PSA consists of 33,572 men who had their PSA level measured between 1994 and 2014, and are not known to have been diagnosed with BPH/LUTS or prostate cancer according to relevant nationwide patient lists. This study was approved by the Data Protection Commission of Iceland and the National Bioethics Committee of Iceland (License No.: VSN-17-026 and VSN-18-029) Written informed consent was obtained from all subjects requited for blood samples. Personal identifiers associated with medical information and blood samples were encrypted with a third-party encryption system.

The UK Biobank BPH/LUTS dataset (accessed under Application Number: 24711) consists of 11,178 men with symptomatic BPH/LUTS, according to hospital-based diagnosis (ICD10 code = N40), as well as 176,541 male controls, not known to have been diagnosed with BPH/LUTS. For the UK GWAS of prostate cancer (ICD10 code = C61) we used 5811 men diagnosed with prostate cancer and 181,908 male controls not know to have prostate cancer.

### Genotyping

The Icelandic BPH/LUTS-, prostate cancer-, and PSA-level GWAS datasets used in the current study are based on whole-genome sequencing, chip genotyping and imputation, aided by long-range phasing of Icelandic population samples^27^. In brief, we whole-genome-sequenced 15,220 Icelanders using Illumina technology (Illumina, San Diego, CA, USA) to an average depth of at least 34×, resulting in the identification of some 94 million variants. Using imputation assisted by long-range haplotype phasing^28, 29^ and after removing variants with imputation information content below 0.8 as well as with an imputed MAF below 0.01%, we successfully inferred the genotypes of 32,463,443 variants in 434,571 Icelanders, of whom 151,677 had been genotyped using the Illumina chip genotyping platform. The remaining 282,894 Icelanders are first- and second-degree relatives of the chip-typed individuals and are imputed by aid of genealogic information.

Genotyping of UKB samples was performed using a custom-made Affymetrix chip, UK BiLEVE Axiom^30^, and with the Affymetrix UK Biobank Axiom array^31^. Imputation was performed by Wellcome Trust Centre for Human Genetics using the Haplotype Reference Consortium (HRC) and the UK10K haplotype resources^32^. This yielded a total of 96 million imputed variants, however only 40 million variants imputed using the HRC reference set were used in this study due to quality issues with the remaining variants.

### GWAS and meta-analysis

Logistic regression assuming an additive model was used to test for association between variants and disease, treating disease status as the response and expected genotype counts from imputation as covariates, and using likelihood ratio test to compute two-sided *P*-values. The association analysis for both the Icelandic and UKB datasets was done using software developed at deCODE genetics^27^. For the Icelandic study group patients and controls are matched on gender, age at inclusion, and information on county of origin within Iceland are included as covariates to adjust for possible population stratification. For the UK datasets, cases and controls are restricted to individuals of genetically confirmed white British origin, and 40 principle components are included in the analysis to adjust for population substructure. The total number, combined in the Icelandic and UK GWASs, of variants tested in our analysis was 42.9 million (with imputation info score > 0.80 in both study groups) in a total of 20,621 patients and 280,541 controls. All variants reported in Table 1 had imputation information score > 0.95, except rs200383755, which had an imputation information score of 0.99 and 0.88 in the Icelandic and UK datasets, respectively. To account for inflation in test statistics due to cryptic relatedness and stratification, we applied the method of LD score regression^26^ to estimate the inflation in the test statistics and adjusted all *P*-values accordingly. The estimated correction factor for BPH/LUTS based on LD score regression was 1.14 for the Icelandic and 1.03 for the UK datasets. For the prostate cancer GWAS, the correction factor was 1.23 and 1.03, respectively, for the Icelandic and the UK datasets.

Variants in the UK imputation dataset were mapped to NCBI Build38 positions and matched to the variants in the Icelandic dataset based on allele variation. The results from the two cohorts were combined using a fixed-effect model in which the cohorts were allowed to have different population frequencies for alleles and genotypes but were assumed to have a common OR and weighted with the inverse of the variance. Heterogeneity (*P*_het_) was tested by comparing the null hypothesis of the effect being the same in all populations to the alternative hypothesis of each population having a different effect using a likelihood ratio test. *I*^2^ lies between 0 and 100% and describes the proportion of total variation in study estimates that is due to heterogeneity.

### Association significance thresholds

The genome-wide significance threshold for the meta-analysis of GWASs of BPH/LUTS in the current study was corrected for all 42,907,111 being tested using a class-specific Bonferroni procedure based on functional weights of classes of variants^16^ (i.e. *P*-value < ((0.05 × weight)/42,907,111). This yielded significance thresholds of: (i) 1.9 × 10^−7^ for 11,465 high-impact variants (comprised of: stop-gained, frameshift, splice acceptor or donor); (ii) 3.9 × 10^−8^ for 197,583 moderate-impact variants (comprised of: missense, splice-region variants and in-frame INDELs); (iii) 3.6 × 10^−9^ for 2,971,445 low-impact variants (comprised of: synonymous variants 3′- and 5′-UTR variants); (iv) 1.8 × 10^−9^ for 5,015,711 intergenic and deep intronic variants overlapping DNase hypersensitivity sites; and (v) 5.9 × 10^−10^ for 34,710,908 other variants (intergenic and deep intronic).

### Conditional analysis

We applied approximate conditional analyses (COJO), implemented in the GCTA software^17^ to the meta-analysis summary statistics to look for additional association signals at each of the genome-wide significant loci. LD between variants was estimated using a set of 8700 whole-genome-sequenced Icelandic individuals. The analysis was restricted to variants within 1 Mb from the index variants and that were present in both the Icelandic and UKB datasets. We tested 14 loci and about 50,000 variants in the conditional analysis and report variants with conditional *P*-value < 1.0 × 10^−6^, obtained using a logistic regression model. The results from GCTA-COJO were verified by conditional analysis using individual genotype data in the Icelandic and UK datasets separately and results presented in Table 1 are obtained by meta-analyzing those results.

### GWAS of serum levels of PSA

To study PSA levels among unaffected men in Iceland, we excluded subjects who had been diagnosed with prostate cancer as recorded by the ICR (between 1955 and 2016) or were known to have undergone TURP between 1983 and 2017. PSA levels were quantile-standardized to a standard normal distribution and corrected for age at measurement, county of birth, and time to death using a generalized additive model with a smooth component on the age and time to death. Most subjects had more than two PSA measurements. Hence, we used the mean of the adjusted and standardized PSA values for each individual.

Quantitative traits were tested for association under the additive model using a linear mixed model implemented in BOLT-LMM^33^. To account for inflation in test statistics due to cryptic relatedness and stratification, we applied the method of LD score regression^26^. For each single-nucleotide polymorphism a classical linear regression using the genotype as an additive covariate and the average PSA value as a response was fitted to test for association.

### Genetic correlation and PRSs

We estimated the genetic correlation between pairs of traits using the cross-trait LD score regression method^26^ and the summary statistics from the Icelandic and UK datasets. In this analysis we used results for about 1.2 million variants, well imputed in both datasets, and for LD information we used pre-computed LD scores for European populations (downloaded from https://data.broadinstitute.org/alkesgroup/LDSCORE/eur_w_ld_chr.tar.bz2). To avoid bias due to overlapping samples, we calculated the genetic correlation between Icelandic GWAS summary statistic for one trait and the UK GWAS summary statistic for the other traits, and the vice versa, and then meta-analyzed those results.

We used PRS analyses of the GWAS results for one trait to investigate its predictive power for another trait. The PRSs were calculated using genotypes for about 630,000 well-imputed autosomal markers. For PRSs generated for Icelandic individuals, we only used chip-typed individuals and we used effect estimated based on GWAS analysis in the UK dataset. We estimated LD between markers using 14,938 phased Icelandic samples and used this LD information to calculate adjusted effect estimates using LDpred^34^. We created several PRSs assuming different fractions of causal markers (the P parameter in LDpred), and selected the PRSs that best predicted the trait itself. These PRSs were then used when we calculate the correlation of the PRS with other traits. The number of individuals belonging to each phenotype group is as follows: serum levels of PSA consisted of 18,929 Icelandic males; prostate cancer consisted of 3464 patients and 43,029 controls; and BPH/LUTS consisted of 5968 patients and 43,594 controls. The correlation between the PRS and traits was calculated using logistic regression in R (v3.5) (http://www.R-project.org) adjusting for year of birth and principle components by including them as covariates in the analysis.

### Bioinformatics analysis

For each lead variant, correlated variants (*r*^2^ > 0.8) were identified using a set of 8700 whole-genome-sequenced Icelandic individuals. These variants were then annotated by intersection with chromatin immunoprecipitation (ChIP) signal data derived from the ENCODE project (www.encodeproject.org); downloaded in pre-processed (MACS v2 algorithm) bigWig format representing analysis of acetylation of lysine K27 of histone H3 (H3K27ac) in 118 different cell types or primary tissues of which 8 were prostate-derived (epithelial cell of prostate, prostate, RWPE1, RWPE2, PC-3, 22Rv1, C4-2B, and VCAP) (see Supplementary Data 1 and 2). The signal *P*-values (derived from MACS v2) were adjusted by the Benjamini-Hochberg procedure to account for multiple hypotheses and the significance threshold set at the 1% false discovery rate. The H3K27ac ChIP-seq data for primary prostate epithelial cells, used in Fig. 2 and Supplementary Data 2, were derived from accession number ENCFF704IWD. DNase hypersensitivity data for the same sample (primary prostate epithelial cells), used in Supplementary Data 2, were derived from accession number ENCFF5450IN. Super-enhancers defined in LNCaP prostate cancer cell line were derived from Hnisz et al.^35^ and the Hi-C data for LNCaP used for defining topologically associated domains are derived from Encode (ENCSR346DCU), downloaded in pre-processed format through the 3D Genome Browser (http://promoter.bx.psu.edu/hi-c/index.html).

A link between the lead variant (and all variants in the corresponding LD class; *r*^2^ > 0.8) and neighboring gene(s) was established using GORpipe analysis tools^36^ and four bioinformatics data sources; Variant effect predictor^37^, JEME^38^, Fantom 5 promoters^39^, and the GTEx project^40^. Differently weighted scores to each data source were given for each LD variant linked to a gene. The weighted scores for each LD class were then summed for each lead variant. A confidence of the link was calculated as follows: (score highest gene − score second highest gene)/score highest gene (see Supplementary Data 1). Transcription factors with significantly impacted binding also reported by Encode were identified using the matchPWM function from Biostrings package in R to enable computational predictions for the impact of single-nucleotide variants on DNA-binding protein occupancy.

## Electronic supplementary material

Supplementary Information

Description of Additional Supplementary Files

Supplementary Data 1

Supplementary Data 2

## Data Availability

The Icelandic population WGS data have been deposited at the European Variant Archive under accession code PRJEB8636. The authors declare that the data supporting the findings of this study are available within the article, its Supplementary Data files, and upon request. The UK Biobank data can be obtained upon application (ukbiobank.ac.uk).
