# Supplementary material for: Genome-wide associations for benign prostatic hyperplasia reveal a genetic correlation with serum levels of PSA
Source: Nat Commun. 2018 Nov 8;9:4568. doi: 10.1038/s41467-018-06920-9 (PMC6224563; doi:10.1038/s41467-018-06920-9)
Supplement: Supplementary file 1 — Supplementary Information [file 41467_2018_6920_MOESM1_ESM.pdf]

Supplementary Information

Genome-wide associations for benign prostatic hyperplasia reveal a genetic correlation with serum levels of PSA

Gudmundsson **et al.**

Supplementary Note 1

Our bioinformatics- and eQTL analyses yielded several interesting findings for the newly discovered BPH/LUTS variants (see Supplementary Data Files 1 and 2,). Regarding the eQTLs search in the GTEx Browser (version 7) we did not identify any significant expression association results in either prostate or bladder tissue. The relevant eQTL results are discussed below and listed in Supplementary Table 4. Below we summarize findings from our bioinformatics analyses for the 23 newly discovered BPH/LUTS loci.

2p16.1

At 2p16.1, two variants, independently associated with BPH/LUTS, were discovered in our study. rs2556378 is located intronic in *BCL11A* which functions as a B-cell proto-oncogene and may play role in leukemogenesis and hematopoiesis. rs10180282 is located in AC007381.3, a long intergenic noncoding RNAs (LincRNA), for which the highest expression is reported in skin and EBV-transformed lymphocytes according to the GTEx Portal.

5p15.33

Among the most strongly associated variants with BPH/LUTS in our study is rs381949. This variant is located intronic in *CLPTM1L* on 5p15.33; a region previously reported to contain multiple variants associated with cancer risk in several different organs. Variants, strongly correlated ($r^2 > 0.85$; see Supplementary Table 8) with rs381949, have been reported to associate with serum levels of PSA[1] and cancer at multiple different sites[2-5]. The conditional analysis revealed a second GWS association signal at 5p15.33, rs2853677, located intronic within *TERT*. This variant has previously been reported to associate with multiple cancer types, being protective for some and at risk for others[6,7]. Interestingly, this variant has no other strongly correlated variant ($r^2>0.75$; according to deCODE's dataset of 8,700 whole-genome sequenced individuals) despite having a MAF of 42% (an average based on the Icelandic and UK controls) and can therefore be considered a credible causal variant for symptomatic BPH/LUTS.

5q22.1

The 5q22.1 variant (rs10054105) is located intergenic between *STARD4* and *NREP* but within the antisense RNA gene *STARD-AS1*. According to UniProt webpage, NREP may have roles in neural function and promotes also axonal regeneration (By similarity). It may also have functions in cellular differentiation (by similarity) and induce differentiation of fibroblast into myofibroblast and myofibroblast ameboid migration. It increases retinoic-acid regulation of lipid-droplet biogenesis (by similarity) and down-regulates the expression of TGFB1 and TGFB2 but not of TGFB3 (by similarity). Whereas STARD4 May be involved in the intracellular transport of sterols or other lipids and it may also bind cholesterol or other sterols (by similarity).

5q31.1

rs677394 at 5q31.1, is located intronic in *C5orf66*, and downstream of *H2AFY*, which encodes a replication-independent histone that is a member of the histone H2A family. It replaces conventional H2A histones in a subset of nucleosomes where it represses transcription and participates in stable X chromosome inactivation. H2AFY is widely expressed but no expression association is reported for rs677394 in the GTEx Portal.

6p22.1

rs200476 is located intergenic on 6p22.1 in the vicinity of a histone gene cluster.

10p12.31

At 10p12.31 we discovered two independent BPH/LUTS signals: rs148678804 and rs7906649. A correlated SNP (rs116940348 with $r^2 = 0.71$) of rs148678804 has been reported to associate with serum levels of PSA[8]. Both of these variants are located upstream of the *DNAJC1* gene and our bioinformatics analysis suggests an effect of both of these variants on a promoter/enhancer region for *DNAJC1*. This gene encodes a membrane bound heat shock protein that binds the molecular chaperone BiP, located in the lumen of the endoplasmic

reticulum. A relatively strong expression of *DNAJC1* is reported in both bladder and prostate tissue according to the GTEx Portal.

10q26.12

The 10q26.12 locus contains three independent BPH/LUTS association signals: rs2981575 located intronic in *FGFR2*, rs4548546 located intronic in *WDR11*, and rs11199879 located intergenic between *WDR11* and *FGFR2* (see Table 1). Members of *WDR11* gene family are involved in a variety of cellular processes, including cell cycle progression, signal transduction, apoptosis, and gene regulation. *FGFR2* is a member of the fibroblast growth factor receptor family and plays an essential role in the regulation of cell proliferation, differentiation, migration and apoptosis. The *FGFR2* intron variant (rs2981575) has previously been shown to associate with BRCA2 associated breast cancer[9]. As for the intergenic variant (rs11199879) a strongly correlated variant, rs10886902 (has $r^2 = 0.99$ with of rs11199879) has been shown to associate with serum levels of PSA[1] and aggressiveness of prostate cancer[10].We checked if any of these three variants were significantly associated with gene expression, based on results in the GTEx Portal. No significant expression association results are reported for any of these variants, after conditioning on the SNP with the most significant eQTL association for the most relevant gene (see Supplementary Table 4).

11p15.5

At 11p15.5, a missense variant in *ODF3*, rs72878024, associates with BPH/LUTS. According to the GTEx Portal, rs72878024 associates with expression levels of *BET1L* in esophagus-muscular tissue after the results have been conditioned on the strongest eQTl marker at this locus (β =-0.33; P = 0.0015, see Supplementary Table 4). ODF3 is a component of sperm flagella outer dense fibers, which are important during sperm movement, whereas BET1L participates in vesicles transport within the Golgi complex. To us this reflects well the diversity of potential biological functions related to a single association signal.

12q24.21

The 12q24.21 locus has two independently associated BPH/LUTS variants. rs2555019 is located intergenic and downstream of *TBX5*, a gene belonging to a gene family that encode transcription factors involved in regulation of embryonic developmental processes. The other variant, rs8853, is correlated (r^2= 0.64) with rs11067228 reported to associate with serum levels of PSA[8] and it is located in the 3'-UTR of *TBX3*, belonging to the same gene family as *TBX5*. Germline mutations in *TBX3* underlie ulnar mammary syndrome, a rare pleiotropic disorder characterized by altered development of upper limbs, apocrine and mammary gland and genital development[11]. Expression levels of *TBX3*, in the GTEx tissue library, are reported to be second and third highest, in bladder- and prostate tissues, respectively. Based on our focused analysis of promoters/enhancer regions in prostate epithelial cells we found the 12q24.12 locus (with rs8853 as a lead variant) to intersect with a super-enhancer and to have a clear tissue-specificity with respect to the H3K27ac mark in prostate-derived cells (Fig. **2a**). Furthermore, based on a recently developed enhancer-gene target resource, referred to as the Joint Effect of Multiple Enhancers (JEME), *TBX3* is the only candidate target gene, in primary prostate tissue samples, linked to this enhancer element.

13q14.3

rs1638703 and rs6561599 on 13q14.3 are both independently associated with BPH/LUTS according to our results. rs1638703 is fully correlated (r^2=1) with rs202346 that has been reported to associate with serum levels of PSA[8] and is located intronic within the non-protein coding gene *DLEU1*, whereas, rs6561599 is located some 5 kb upstream of *RNASEH2B*. The protein encoded by this gene is the non-catalytic B-subunit of RNase H2 endonuclease complex, which is thought to play a role in nucleic acid metabolism to preserve genome stability and to prevent immune activation[12]. Our focused analysis (with rs6561599 as a lead variant) of promoters/enhancers revealed a tissue-specific promoter region for *RNASEH2B*, wherein the H3K27ac mark was particularly prevalent in prostate-derived cells (see Fig. 2b).

17q12

At 17q12, rs11651052 is located intronic in *HNF1B* and has previously been reported to associate with increased risk of prostate cancer and serum levels of PSA (rs11651052 has r^2=0.91 with rs4430796 Ref[13], see Supplementary Table 8).

18q11.2

The 18q11.2 BPH/LUTS locus contains one variant (rs9958656) that is located intergenic between *GATA6* and *CTAGE1* (Cutaneous T-Cell Lymphoma-Associated Antigen 1). The *GATA6* gene is a member of a small family of zinc finger transcription factors that play an important role in the regulation of cellular differentiation and organogenesis during vertebrate development. The GTEx Portal reports the highest expression levels of *GATA6* to be in the ovary and *CTAGE1* is only reported to be expressed in testis.

19q12

At 19q12, the BPH/LUTS variant, rs11084596, is located upstream of *THEG5* (testis highly expressed protein 5) a gene which function is unknown.

20q13.33

The 20q13.33 locus contains two independent variants associated with symptomatic BPH/LUTS. One of these variants, rs200383755_C, is a missense variant (p.Ser19Trp) in the *GATA5* gene. In our combined study group this variant has an average minor allele frequency in controls of 0.9%, and a strong protective effect against symptomatic BPH/LUTS, with an $OR_{conditioned} = 0.67$ and $P_{conditioned} = 3.2\times10^{-9}$ (Table 1). The protein encoded by this gene is a transcription factor that contains two GATA-type zinc fingers and is required during cardiovascular development[14]. According to the GTEx Portal, *GATA5* has the highest expression in bladder but its expression is also relatively high in prostate tissue, ranking seventh from the top. The other independently associated variant at 20q13.33 is rs6061244_C ($OR_{conditioned} = 0.94$ and $P_{conditioned} = 5.7\times10^{-8}$; see Table 1), located intronic in *GATA5* and it has no strongly correlated variants ($r^2 < 0.75$) and can therefore, be considered a credible risk variant.

Supplementary Figures

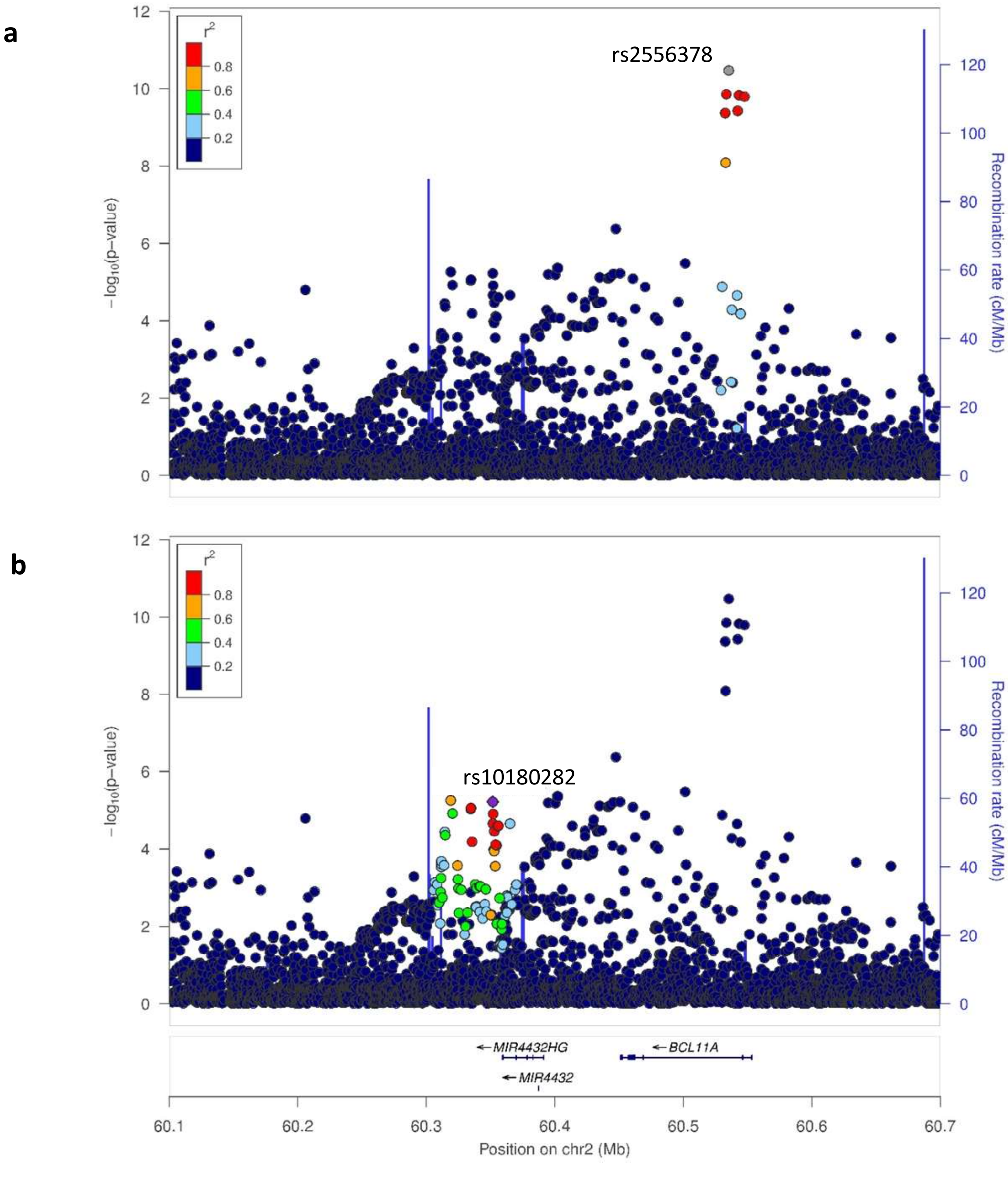

Supplementary Figure 1. (See figure legends below)

c

d

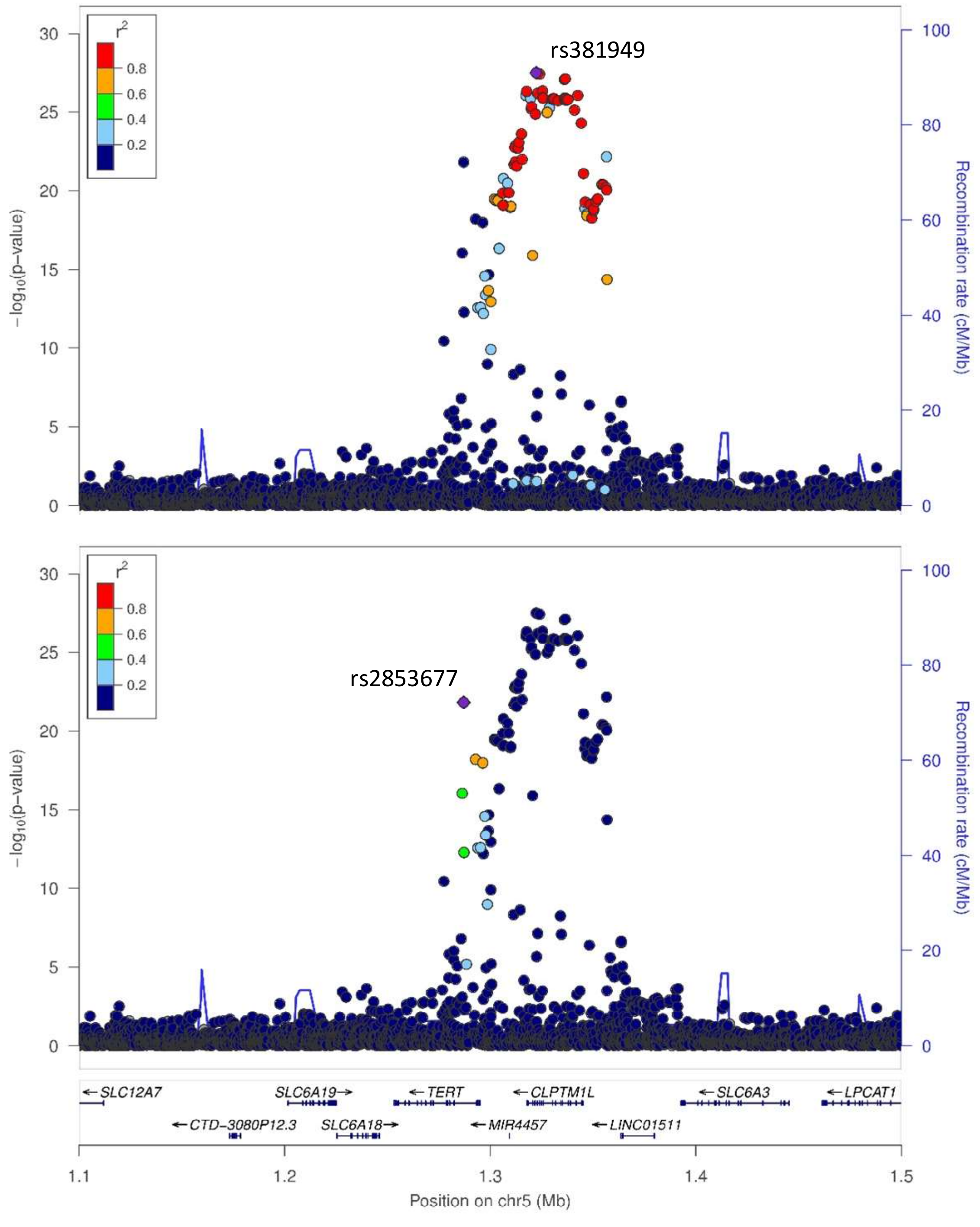

Supplementary Figure 1. Cont. (See figure legends below)

e

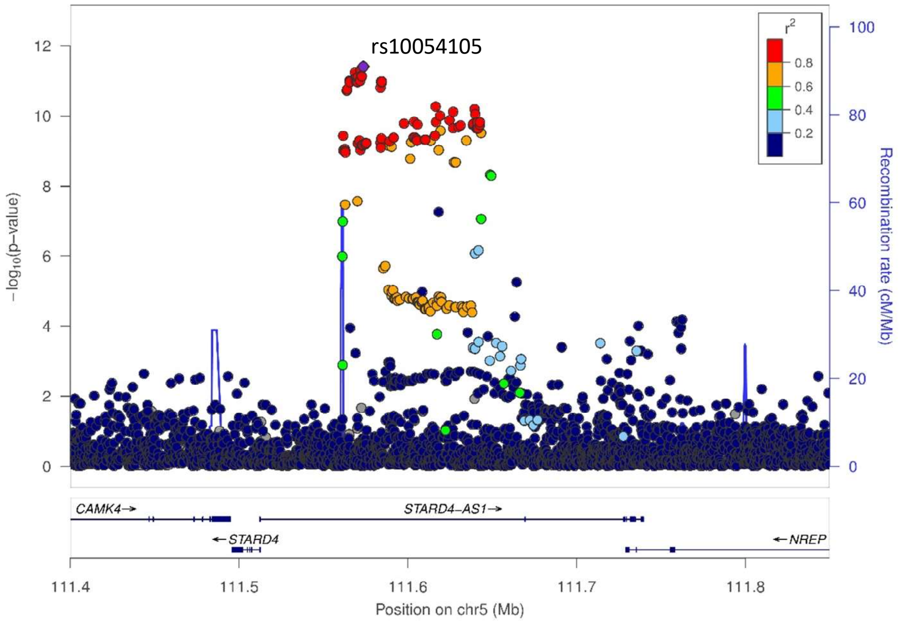

f

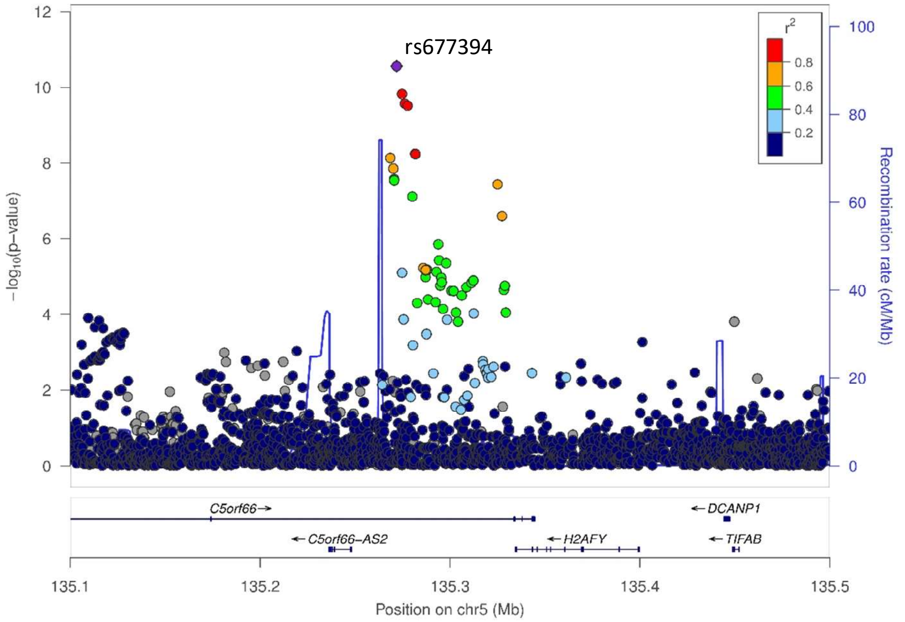

Supplementary Figure 1. Cont. (See figure legends below).

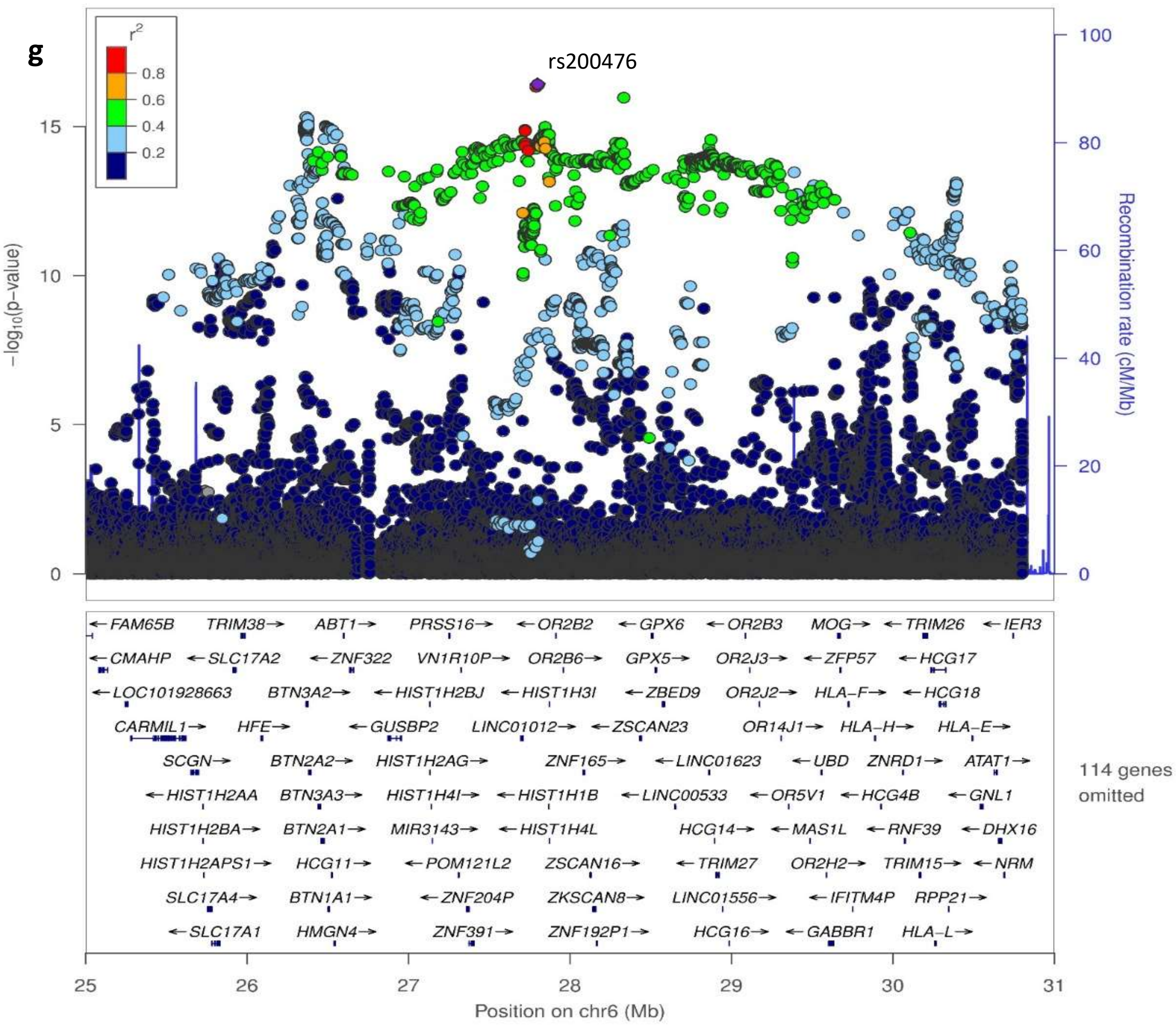

Supplementary Figure 1. Cont. (See figure legends below).

h

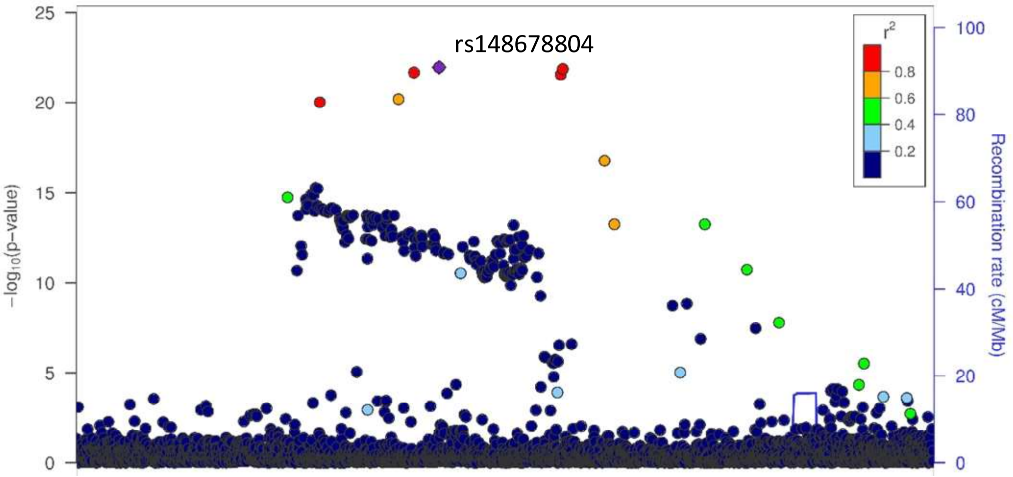

i

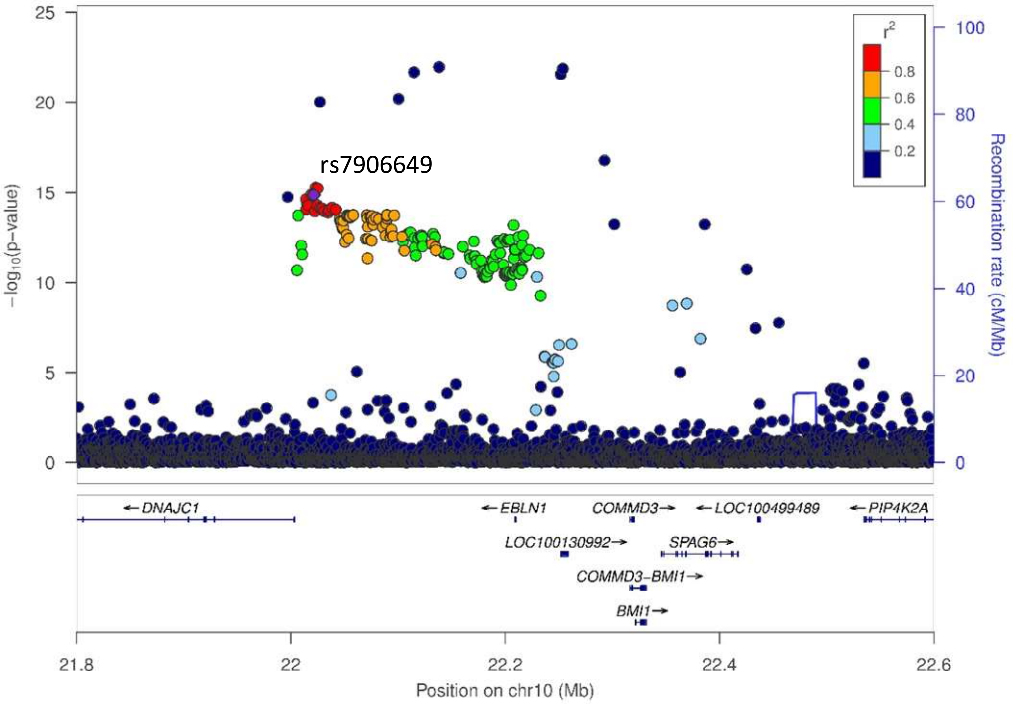

Supplementary Figure 1. Cont. (See figure legends below).

j

k

l

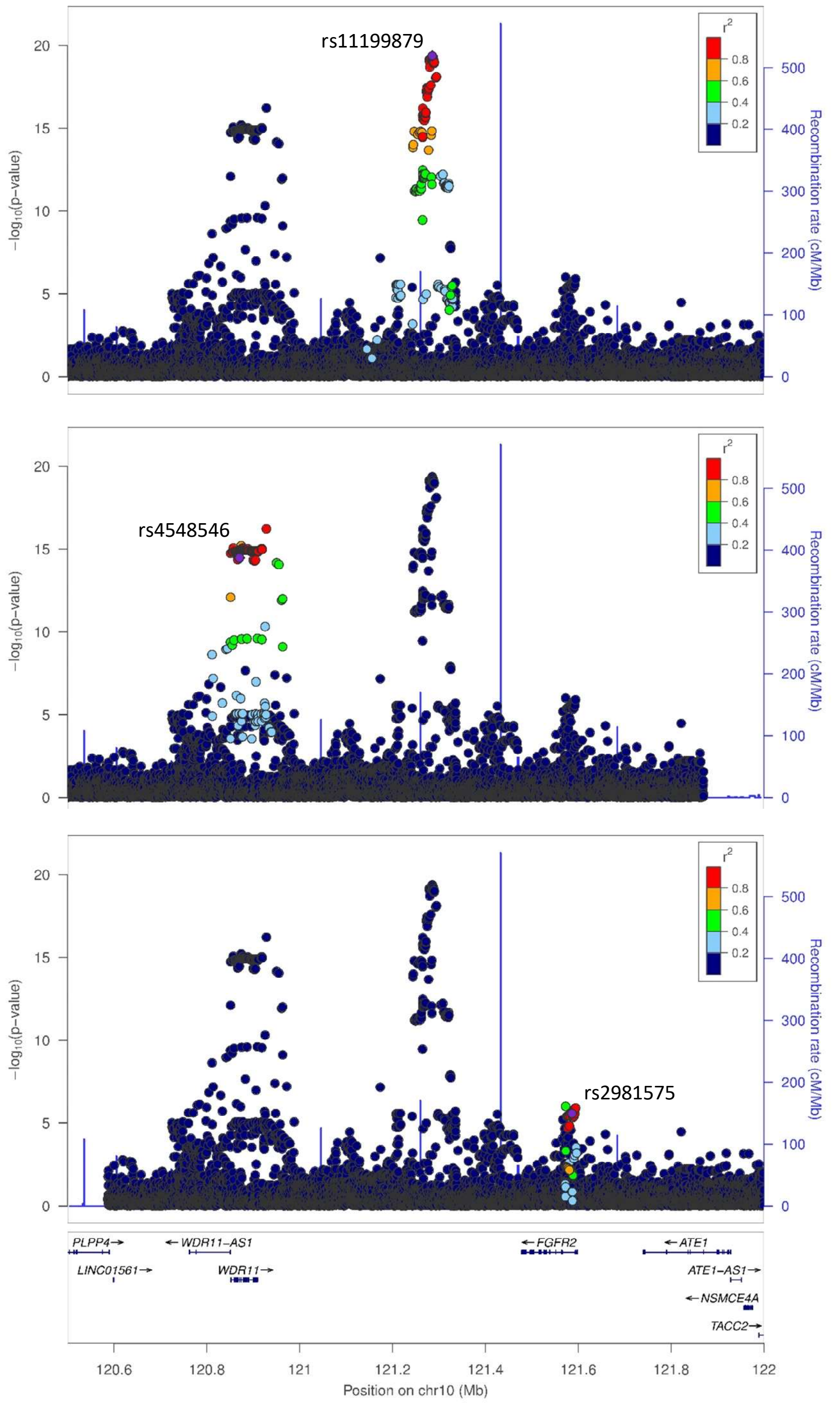

Supplementary Figure 1. Cont. (See figure legends below).

m

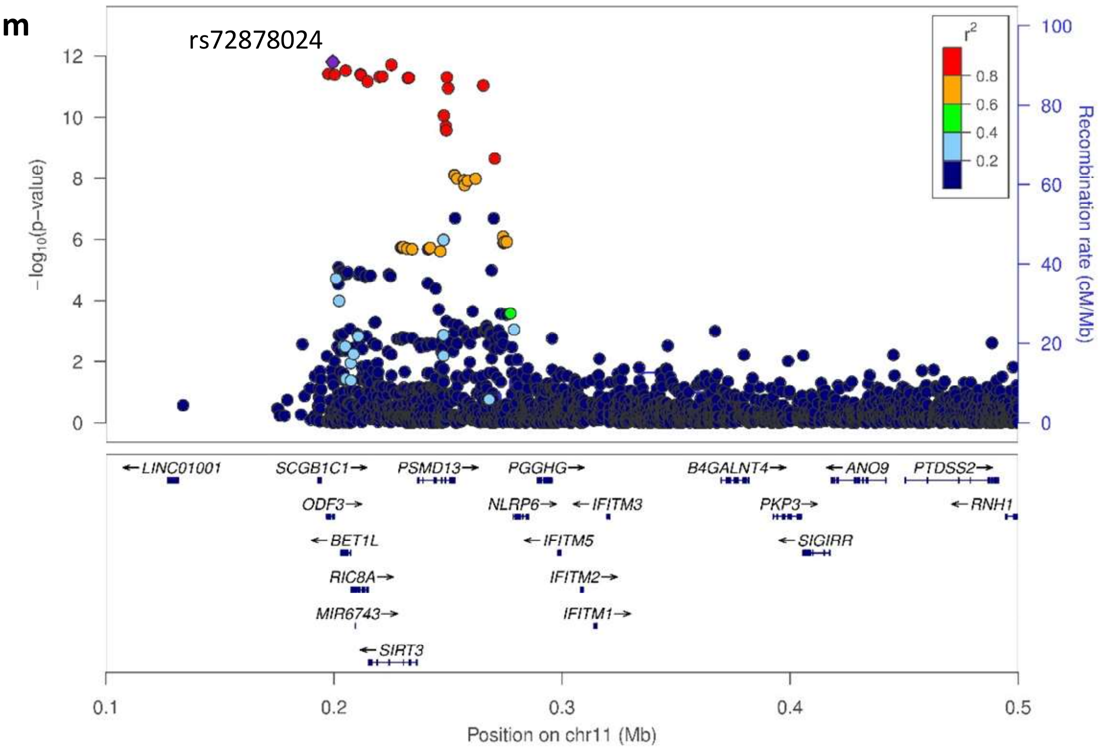

Supplementary Figure 1. Cont. (See figure legends below).

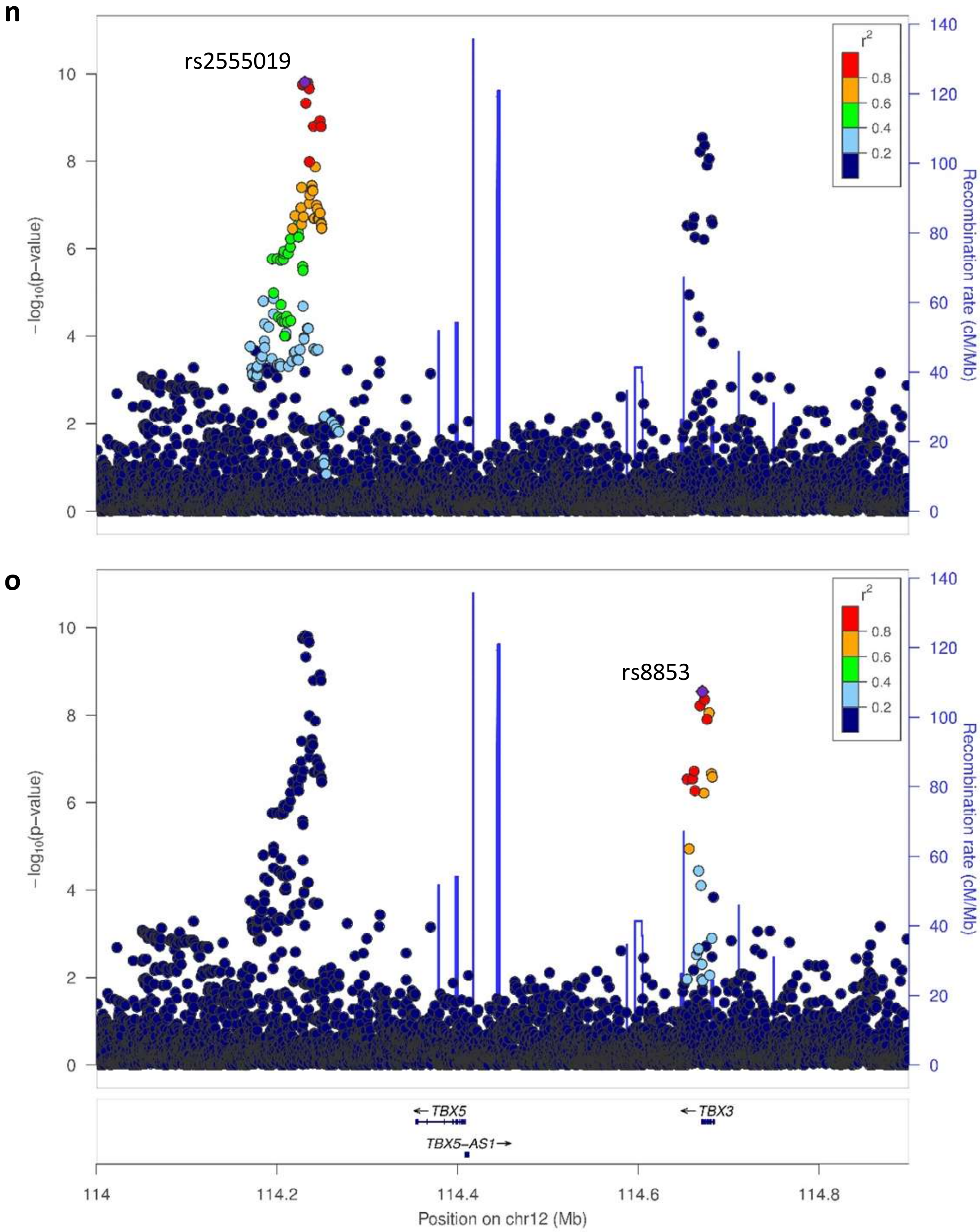

Supplementary Figure 1. Cont. (See figure legends below).

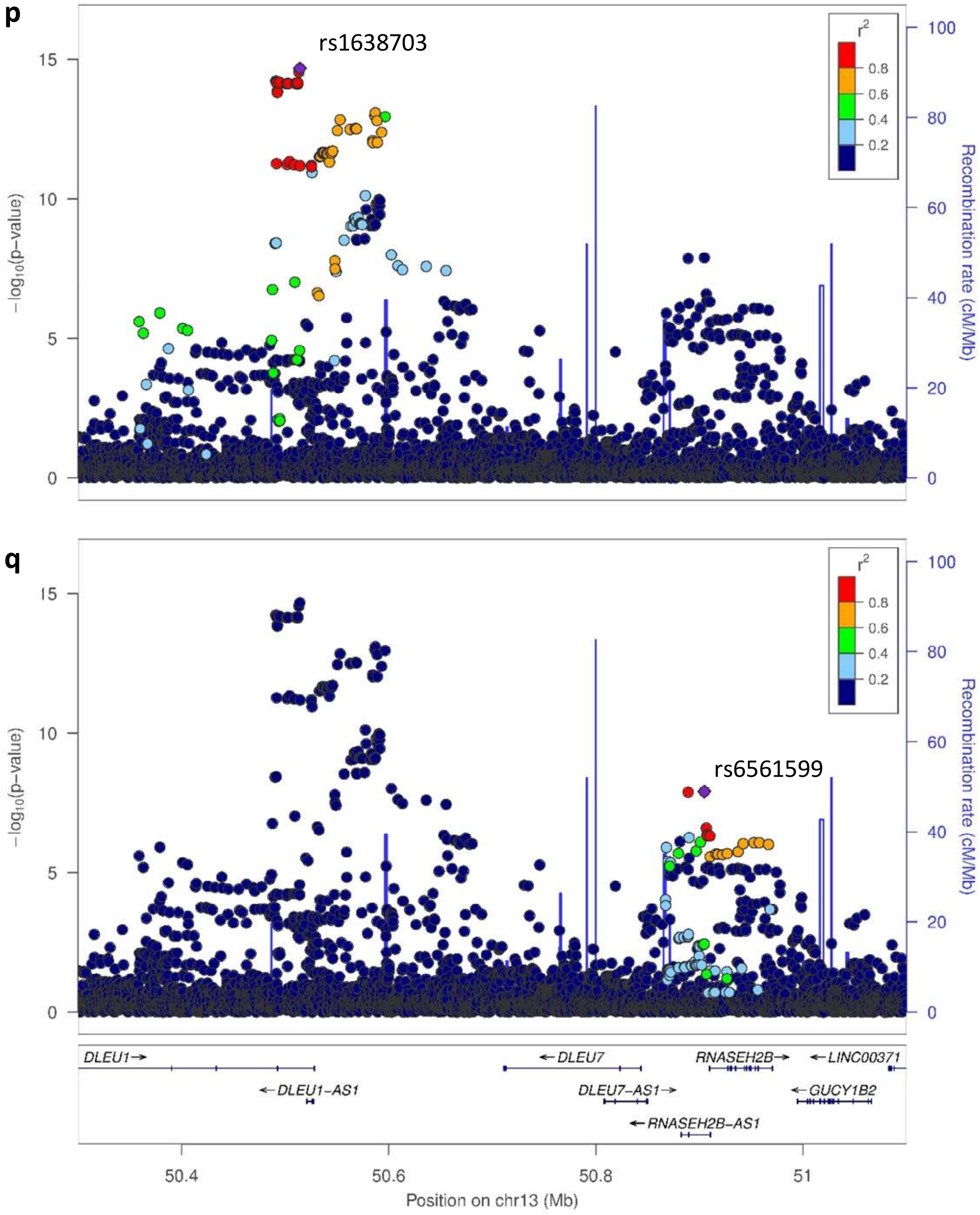

Supplementary Figure 1. Cont. (See figure legends below).

r

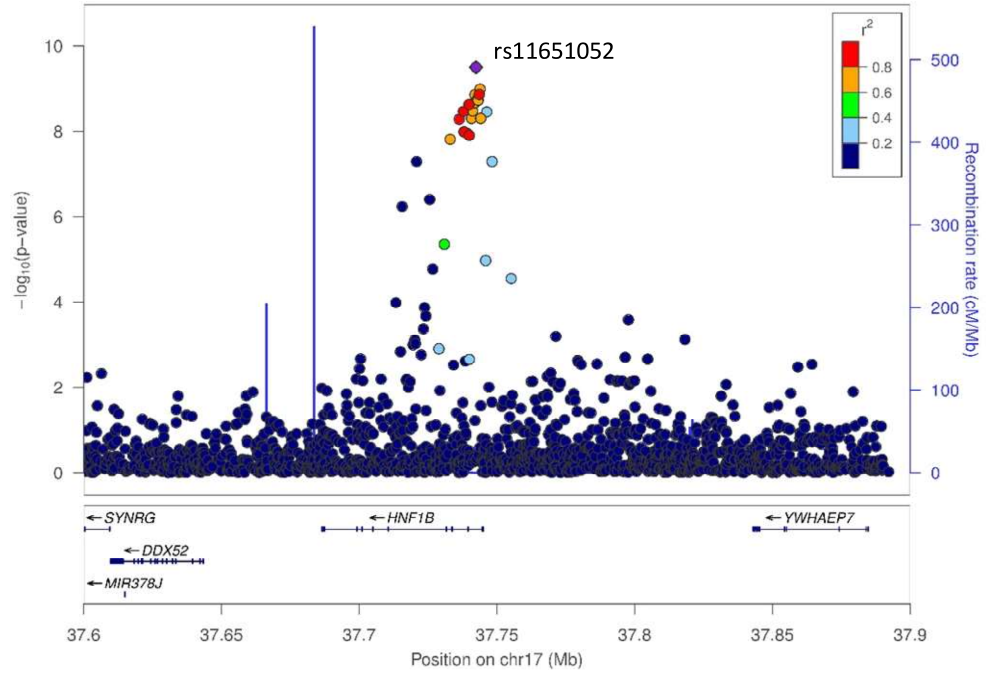

Supplementary Figure 1. Cont. (See figure legends below).

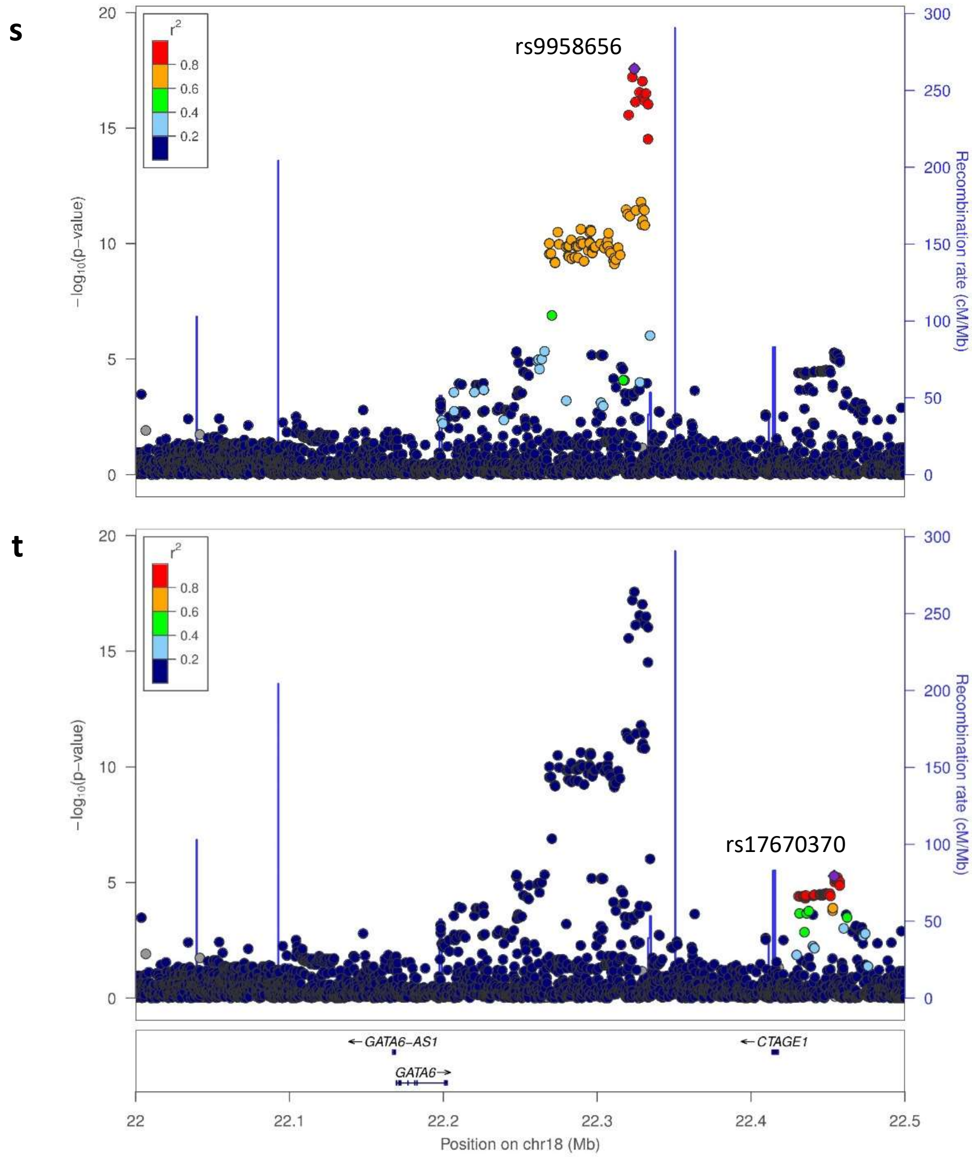

Supplementary Figure 1. Cont. (See figure legends below).

u

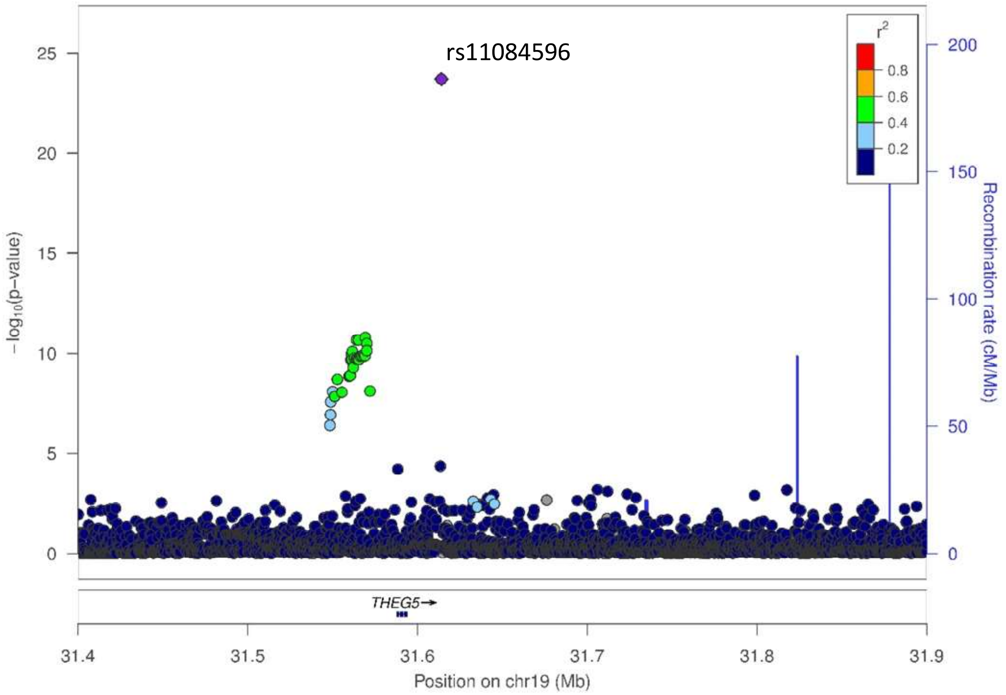

Supplementary Figure 1. Cont. (See figure legends below).

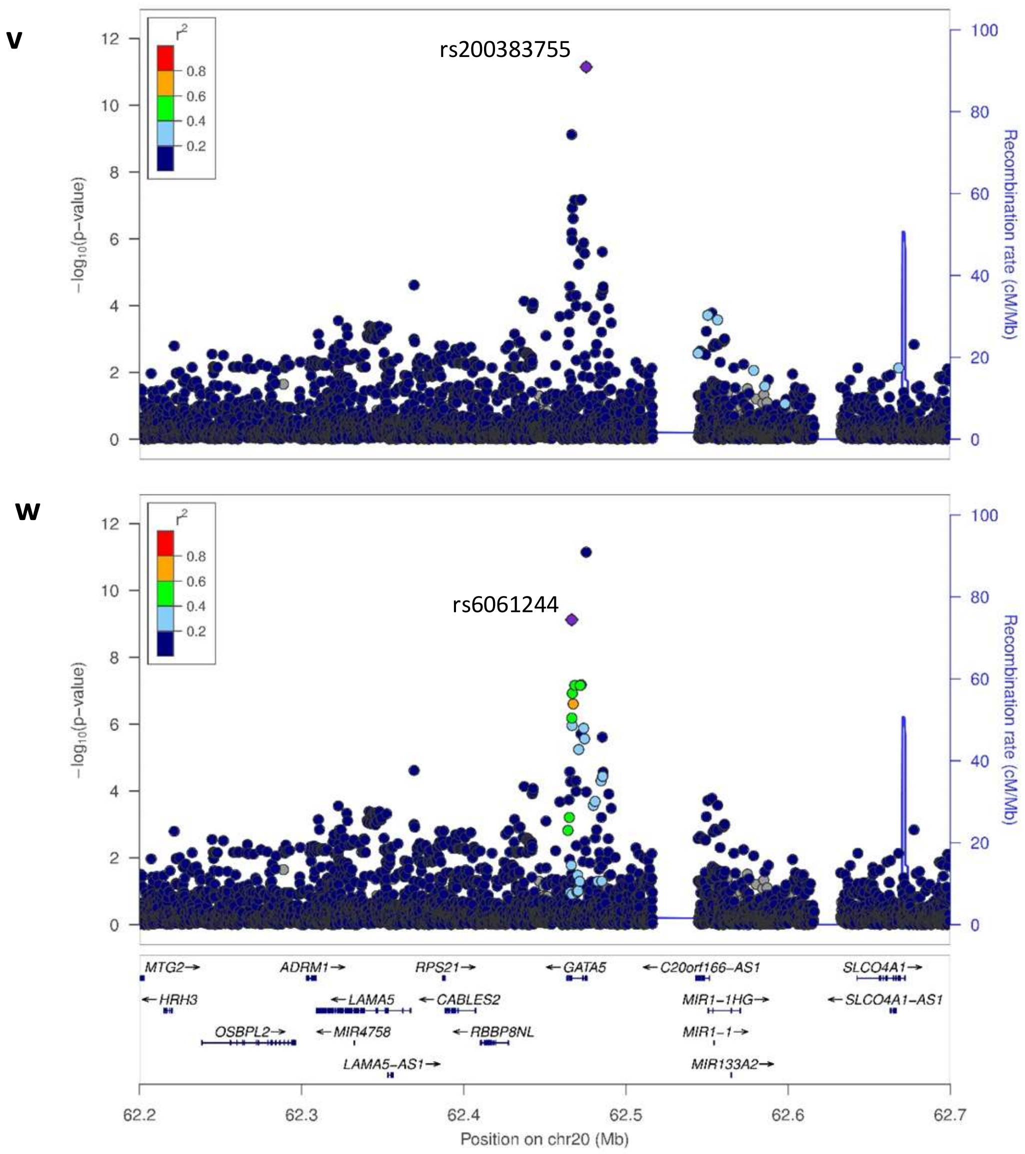

Supplementary Figure 1. Cont. Regional association plot for variants with imputation information score ≥0.80 and located at one of the BPBH/LUTS risk loci reported in main text. Shown are the negative log10-transformed P-values from the meta-analysis of GWASs in Iceland and the UK. The risk loci are located on: 2p16.1 (**a** and **b**), 5p15.33 (**c** and **d**), 5q22.1 (**e**), 5q31.1 (**f**), 6p22.1 (**g**), 10p12.31 (**h** and **i**), 10q26.12 (**j**, **k**, and **l**), 11p15.5 (**m**), 12q24.21 (**n** and **o**), 13q14.3 (**p** and **q**), 17q12 (**r**), 18q11.2 (**s** and **t**), 19q12 (**u**), and 20q13.33 (**v** and **w**). The lead marker at each locus is denoted by a purple circle and other circle-colors indicate linkage disequilibrium (LD) with lead marker (LD is represented as the correlation coefficient (r^2) calculated based on the Icelandic whole-genome sequencing dataset. The blue line shows the recombination rate from Phase 2 HapMap estimated from phased haplotypes in HapMap Release 22 (Ref.[15]) estimated from the CEU, YRI and JPT+CHB populations, and mapped onto NCBI hg38, Build38. The plot was created using a standalone version of the LocusZoom software[16]

a)

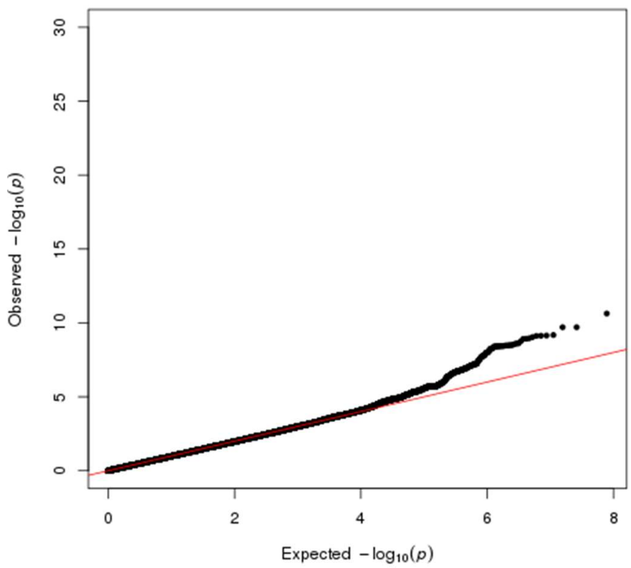

b)

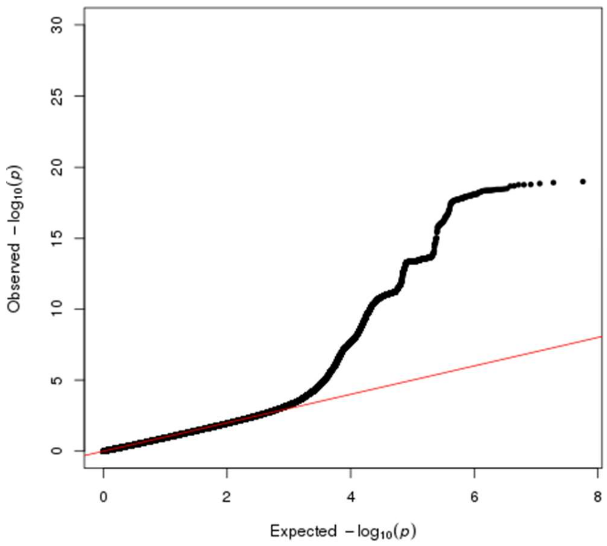

Supplementary Figure 2. A quantile-quantile plot (QQ-plot) showing chi-square statistics corrected for genomic control and with high (>0.8) imputation information content from the GWAS of BPH/LUTS in **a)** Iceland and **b)** the UK. The red diagonal line represents expected distribution assuming no inflation of the chi-square statistics.

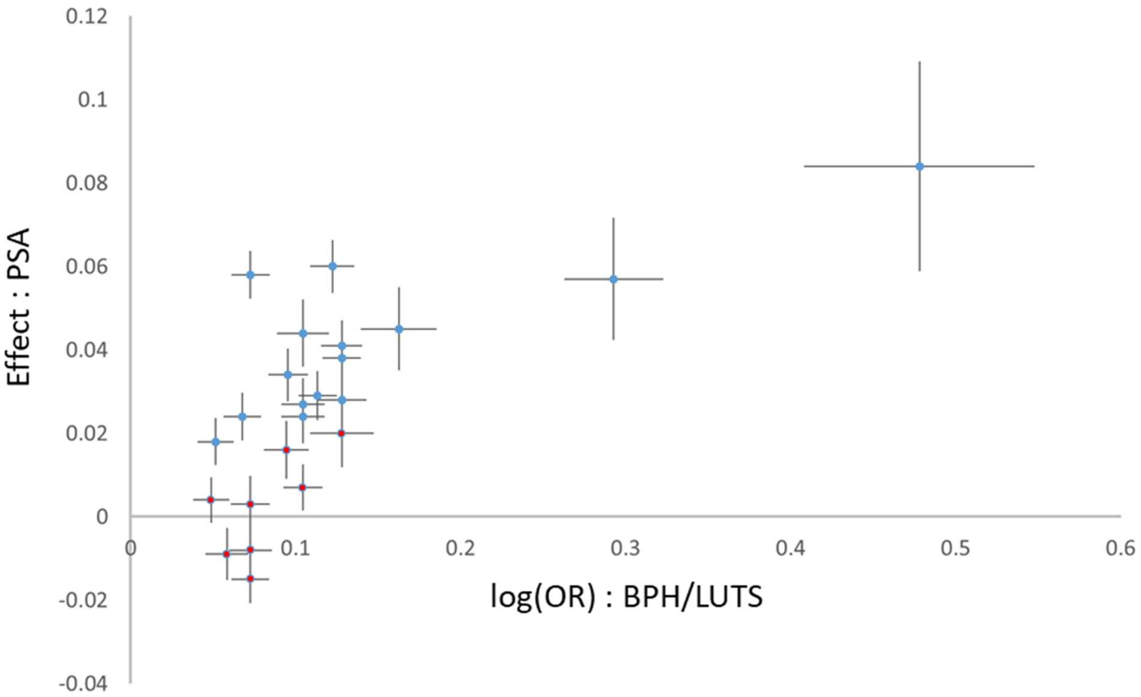

Supplementary Figure 3. Comparison of the effect of variants on PSA-levels vs. on benign prostatic hyperplasia/lower urinary tract symptoms (BPH/LUTS). Results shown are for variants reported in main text and that are genome-wide significantly associate with BPH/LUTS. On the y-axis are their effect estimates (β) from the GWAS of PSA-levels in Iceland and on the x-axis is the natural logarithm of the odds ratio (log(OR)) from the meta-analysis of the GWASs of BPH/LUTS in Iceland and the UK. The blue dots denote BPH/LUTS-variants significantly associated with PSA-levels at a Bonferroni correction of $P < 0.0022$ and red dots denote variants not surpassing the Bonferroni significance threshold. Error bars denote standard error. The depicted data is also reported in Supplementary Table 7.

Supplementary Table 1. Results from the GWAS of symptomatic BPH/LUTS and the conditional analysis for loci with multiple variants.

Locus	Marker (EA/OA)	Covariate	Annotation / Nearby gene(s)	EAF	P_{het} / I^2 (%)	Iceland		UK		Combined	
						OR (95% c.i.)	P-value	OR (95% c.i.)	P-value	OR (95% c.i.)	P-value
2p16.1	rs2556378 (T/G)	rs10180282	Intron variant / *BCL11A*	0.154	0.37/0	1.09 (1.04, 1.15)	9.2E-04	1.13 (1.09, 1.17)	6.2E-10	1.12 (1.08, 1.15)	3.4E-12
2p16.1	rs10180282* (T/C)	rs2556378	Intergenic variant / *BCL11A*	0.456	0.58/0	1.07 (1.03, 1.11)	8.7E-04	1.05 (1.02, 1.08)	2.5E-04	1.06 (1.03, 1.08)	8.7E-07
5p15.33	rs381949 (A/G)	rs2853677	Intron variant / *CLPTM1L*	0.415	0.86/0	0.90 (0.86, 0.93)	7.8E-08	0.90 (0.87, 0.93)	1.1E-12	0.90 (0.88, 0.92)	4.9E-19
5p15.33	rs2853677* (G/A)	rs381949	Intron variant / *TERT*	0.421	0.44/0	1.07 (1.03, 1.12)	5.0E-04	1.09 (1.06, 1.13)	6.0E-10	1.09 (1.06, 1.11)	1.7E-12
5q22.1	rs10054105 (G/T)	na	Intergenic variant / *STARD4*	0.213	0.65/0	0.91 (0.87, 0.96)	9.2E-05	0.90 (0.87, 0.93)	7.8E-09	0.91 (0.88, 0.93)	3.5E-12
5q31.1	rs677394 (G/C)	na	Intron variant / *C5orf66, H2AFY*	0.123	0.034/78	0.92 (0.87, 0.97)	2.9E-03	0.85 (0.81, 0.90)	2.7E-10	0.88 (0.85, 0.92)	2.9E-11
6p22.1	rs200476 (T/A)	na	Intergenic variant / *HIST1H2BL*	0.162	0.23/30	0.85 (0.80, 0.90)	7.9E-08	0.89 (0.85, 0.92)	4.4E-11	0.88 (0.85, 0.90)	3.9E-17
10p12.31	rs148678804 (A/G)	rs7906649	Intergenic variant / *DNAJC1*	0.035	0.17/48	1.20 (1.09, 1.33)	1.8E-04	1.31 (1.21, 1.42)	1.5E-11	1.27 (1.19, 1.35)	3.0E-14
10p12.31	rs7906649* (G/A)	rs148678804	Intergenic variant / *EBLN1*	0.286	0.71/0	1.06 (1.02, 1.11)	6.0E-03	1.07 (1.04, 1.11)	1.0E-05	1.07 (1.04, 1.10)	2.1E-07
10q26.12	rs11199879 (C/T)	rs4548546 and rs2981575	Intergenic variant / *FGFR2*	0.252	0.021/81	1.10 (1.05, 1.14)	1.9E-05	1.17 (1.13, 1.21)	4.1E-20	1.14 (1.11, 1.17)	5.7E-23
10q26.12	rs4548546* (T/C)	rs11199879 and rs2981575	Intron variant / *WDR11*	0.310	0.20/40	1.08 (1.04, 1.13)	2.9E-04	1.12 (1.09, 1.15)	6.7E-14	1.11 (1.08, 1.13)	2.0E-16
10q26.12	rs2981575* (G/A)	rs11199879 and rs4548546	Intron variant / *FGFR2*	0.427	0.97/0	0.94 (0.90, 0.97)	1.1E-03	0.94 (0.91, 0.97)	1.5E-05	0.94 (0.92, 0.96)	6.0E-08
11p15.5	rs72878024 (A/G)	na	Missense variant / *ODF3*	0.080	0.20/40	0.82 (0.77, 0.88)	7.4E-08	0.87 (0.83, 0.92)	1.7E-06	0.85 (0.82, 0.89)	1.4E-12
12q24.21	rs2555019 (T/C)	rs8853	Intergenic variant / *TBX5*	0.456	0.82/0	0.92 (0.89, 0.96)	4.1E-05	0.93 (0.90, 0.95)	1.3E-07	0.93 (0.91, 0.95)	2.4E-11
12q24.21	rs8853* (C/T)	rs2555019	3-prime UTR variant / *TBX3*	0.494	0.75/0	1.07 (1.03, 1.11)	9.8E-04	1.07 (1.04, 1.10)	3.7E-07	1.07 (1.05, 1.10)	1.4E-09
13q14.3	rs1638703 (C/G)	rs6561599	Intron variant / *DLEU1*	0.256	0.57/0	1.09 (1.04, 1.14)	8.7E-05	1.11 (1.07, 1.14)	2.3E-10	1.10 (1.07, 1.13)	1.1E-13
13q14.3	rs6561599* (C/G)	rs1638703	Upstream gene variant / *RNASEH2B*	0.371	1.0/0	0.94 (0.90, 0.98)	2.3E-03	0.94 (0.91, 0.97)	2.3E-05	0.94 (0.92, 0.96)	1.8E-07
17q12	rs11651052 (A/G)	na	Intron-variant / *HNF1B*	0.470	0.24/29	0.95 (0.91, 0.98)	5.5E-03	0.92 (0.89, 0.95)	8.1E-09	0.93 (0.91, 0.95)	3.2E-10
18q11.2	rs9958656 (T/C)	rs17670370	Intergenic variant / *GATA6*	0.430	1.0/0	1.11 (1.07, 1.15)	1.5E-07	1.11 (1.08, 1.14)	5.4E-13	1.11 (1.08, 1.13)	4.3E-19
18q11.2	rs17670370* (G/T)	rs9958656	Intergenic variant / CTAGE1	0.262	0.24/28	1.05 (1.01, 1.09)	2.7E-02	1.08 (1.05, 1.12)	9.9E-07	1.07 (1.04, 1.10)	1.6E-07
19q12	rs11084596 (C/T)	na	Intergenic variant / *THEG5*	0.356	0.34/0	0.90 (0.86, 0.93)	2.4E-07	0.88 (0.85, 0.90)	9.6E-19	0.88 (0.86, 0.90)	2.1E-24
20q13.33	rs200383755 (C/G)	rs6061244	Missense variant / *GATA5*	0.0091	0.53/0	0.65 (0.54, 0.78)	3.0E-06	0.70 (0.58, 0.85)	2.2E-04	0.67 (0.59, 0.77)	3.2E-09
20q13.33	rs6061244* (C/G)	rs200383755	Intron variant / *GATA5*	0.386	0.16/49	0.96 (0.92, 1.00)	0.046	0.93 (0.90, 0.95)	1.6E-07	0.94 (0.92, 0.96)	5.7E-08

Shown is the effect allele (EA), the other allele (OA), the simple average effect allele population frequency (EAF), the allelic odds ratio (OR) for the effect allele with upper and lower 95% confidence intervals (c.i.) and the two-sided P value for association testing between variants and disease which was performed using the likelihood ratio statistic. Results from the two study groups were combined using a Mantel-Haenszel model (see Methods). Annotation is according to Variant Effect Predictor (VEP). Shown are also the P value for the heterogeneity (P_{het}) between the two study groups and the heterogeneity (cont. on next page)

statistic (I^2) representing the fraction of variability due to heterogeneity between study groups. rs200383755 had an imputation information score of 0.99 and 0.88 in the Icelandic and UK datasets, respectively. All other markers listed had imputation information score > 0.95. Results for markers pertaining to loci with more than one association signal are shown after conditioning on a relevant covariate. Markers at loci with no additional association signal do not have any applicable covariate (na) and the results are the unconditioned association result from the GWAS of symptomatic BPH/LUTS.

*Markers discovered in the conditional analysis.

Supplementary Table 2. Results from a GWAS of symptomatic BPH/LUTS in Iceland and the UK.

Locus	Marker (EA/OA)	Position (B38)	Annotation / Nearby gene(s)	EAF	P_{het} / I^2 (%)	Iceland		UK		Combined	
						OR (95% c.i.)	P-value	OR (95% c.i.)	P-value	OR (95% c.i.)	P-value
**a) Variants discovered in the unconditional GWAS analysis**											
2p16.1	rs2556378 (T/G)	60,535,367	Intron variant / *BCL11A*	0.154	0.31/2	1.08 (1.03, 1.14)	2.5E-03	1.12 (1.08, 1.17)	2.1E-09	1.11 (1.08, 1.14)	3.3E-11
5p15.33	rs381949 (A/G)	1,322,353	Intron variant / *CLPTM1L*	0.415	0.82/0	0.88 (0.85, 0.92)	2.0E-10	0.88 (0.85, 0.90)	1.7E-19	0.88 (0.86, 0.90)	2.3E-28
5q22.1	rs10054105 (G/T)	111,573,636	Intergenic variant / *STARD4*	0.213	0.65/0	0.91 (0.87, 0.96)	9.2E-05	0.9 (0.87, 0.93)	7.8E-09	0.91 (0.88, 0.93)	3.5E-12
5q31.1	rs677394 (G/C)	135,271,869	Intron variant / *C5orf66, H2AFY*	0.123	0.034/78	0.92 (0.87, 0.97)	2.9E-03	0.85 (0.81, 0.90)	2.7E-10	0.88 (0.85, 0.92)	2.9E-11
6p22.1	rs200476 (T/A)	27,800,570	Intergenic variant / *HIST1H2BL*	0.162	0.23/30	0.85 (0.80, 0.90)	7.9E-08	0.89 (0.85, 0.92)	4.4E-11	0.88 (0.85, 0.90)	3.9E-17
10p12.31	rs148678804 (A/G)	22,138,360	Intergenic variant / *DNAJC1*	0.035	0.16/50	1.27 (1.15, 1.39)	6.3E-07	1.38 (1.28, 1.49)	1.0E-17	1.34 (1.26, 1.41)	1.0E-22
10q26.12	rs11199879 (C/T)	121,285,698	Intergenic variant / *FGFR2*	0.252	0.0092/85	1.08 (1.04, 1.13)	3.2E-04	1.16 (1.12, 1.20)	8.0E-19	1.13 (1.10, 1.16)	3.5E-20
11p15.5	rs72878024 (A/G)	199,492	Missense variant / *ODF3*	0.080	0.20/40	0.82 (0.77, 0.88)	7.4E-08	0.87 (0.83, 0.92)	1.7E-06	0.85 (0.82, 0.89)	1.4E-12
12q24.21	rs2555019 (T/C)	114,230,813	Intergenic variant / *TBX5*	0.456	0.93/0	0.93 (0.89, 0.96)	9.9E-05	0.93 (0.90, 0.96)	3.4E-07	0.93 (0.91, 0.95)	1.4E-10
13q14.3	rs1638703 (C/G)	50,514,220	Intron variant / *DLEU1*	0.256	0.43/0	1.09 (1.05, 1.14)	5.1E-05	1.12 (1.08, 1.15)	6.2E-12	1.11 (1.08, 1.14)	2.0E-15
17q12	rs11651052 (A/G)	37,742,390	Intron variant / *HNF1B*	0.470	0.24/29	0.95 (0.91, 0.98)	5.5E-03	0.92 (0.89, 0.95)	8.1E-09	0.93 (0.91, 0.95)	3.2E-10
18q11.2	rs9958656 (T/C)	22,324,181	Intergenic variant / *GATA6*	0.430	0.97/0	1.11 (1.07, 1.15)	2.0E-07	1.11 (1.08, 1.14)	2.9E-12	1.11 (1.08, 1.13)	3.1E-18
19q12	rs11084596 (C/T)	31,614,073	Intergenic variant / *THEG5*	0.356	0.34/0	0.9 (0.86, 0.93)	2.4E-07	0.88 (0.85, 0.90)	9.6E-19	0.88 (0.86, 0.90)	2.1E-24
20q13.33	rs200383755 (C/G)	62,475,466	Missense variant / *GATA5*	0.0091	0.77/0	0.63 (0.52, 0.75)	5.7E-07	0.6 (0.49, 0.74)	2.7E-06	0.62 (0.54, 0.71)	7.3E-12
**b) Variants discovered in the conditional GWAS analysis**											
2p16.1	rs10180282 (T/C)	60,351,708	Intergenic variant / *BCL11A*	0.456	0.55/0	1.06 (1.02, 1.10)	1.5E-03	1.05 (1.02, 1.08)	1.0E-03	1.05 (1.03, 1.08)	5.9E-06
5p15.33	rs2853677 (G/A)	1,287,079	Intron variant / *TERT*	0.421	0.25/23	1.1 (1.06, 1.14)	1.2E-06	1.13 (1.10, 1.16)	1.1E-17	1.12 (1.09, 1.15)	1.5E-22
10p12.31	rs7906649 (G/A)	22,021,369	Intergenic variant / *EBLN1*	0.286	0.45/0	1.09 (1.05, 1.14)	3.4E-05	1.11 (1.08, 1.15)	6.7E-12	1.11 (1.08, 1.13)	1.5E-15
10q26.12	rs4548546 (T/C)	120,870,067	Intron variant / *WDR11*	0.310	0.11/62	1.07 (1.03, 1.12)	1.3E-03	1.12 (1.08, 1.15)	1.7E-13	1.1 (1.08, 1.13)	3.2E-15
10q26.12	rs2981575 (G/A)	121,586,602	Intron variant / *FGFR2*	0.427	0.86/0	0.95 (0.91, 0.99)	6.8E-03	0.94 (0.92, 0.97)	1.3E-04	0.95 (0.92, 0.97)	2.7E-06
12q24.21	rs8853 (C/T)	114,671,102	3-prime UTR variant / *TBX3*	0.494	0.54/0	1.06 (1.02, 1.10)	2.8E-03	1.08 (1.05, 1.11)	3.0E-07	1.07 (1.05, 1.09)	3.6E-09
13q14.3	rs6561599 (C/G)	50,904,782	Upstream gene variant / *RNASEH2B*	0.371	1.0/0	0.93 (0.90, 0.97)	7.0E-04	0.93 (0.91, 0.96)	5.3E-06	0.93 (0.91, 0.96)	1.4E-08
18q11.2	rs17670370 (G/T)	22,454,054	Intergenic variant / *CTAGE1*	0.262	0.37/0	1.04 (1.00, 1.09)	0.042	1.07 (1.04, 1.11)	3.0E-05	1.06 (1.03,1.09)	5.3E-06
20q13.33	rs6061244 (C/G)	62,466,597	Intron variant / *GATA5*	0.386	0.30/6	0.94 (0.91, 0.98)	4.8E-03	0.92 (0.89, 0.95)	2.8E-08	0.93 (0.91, 0.95)	8.1E-10

Position is according to Build 38 (hg19) of the reference genome. Shown is the effect allele (EA), the other allele (OA), the simple average effect allele population frequency (EAF), the allelic odds ratio (OR) for the effect allele with upper and lower 95% confidence intervals (c.i.) and the P value for association testing between variants and disease which was performed using logistic regression and the likelihood ratio statistic. Results from the two study groups were combined using a Mantel-Haenszel model (see Methods). Annotation is according to Variant Effect Predictor (VEP). Shown are also the P value for the heterogeneity (Phet) between the two study groups and the heterogeneity statistic (I^2) representing the fraction of variability due to heterogeneity between study groups. rs200383755 had an imputation information score of 0.99 and 0.88 in the Icelandic and UK datasets, respectively. All other markers listed had imputation information score >0.95.

Supplementary Table 3. Pairwise correlation between variants at BPH/LUTS loci with two or more independent association signals.

Locus	*Marker-1*	*Marker-2*	r^2	*D'*
2p16.1	rs10180282	rs2556378	0.0019	0.11
5p15.33	rs2853677	rs381949	0.049	0.31
10p12.31	rs7906649	rs148678804	0.098	1
10q26.12	rs4548546	rs11199879	0.0025	0.12
10q26.12	rs4548546	rs2981575	<0.0001	0.002
10q26.12	rs11199879	rs2981575	0.013	0.16
12q24.21	rs2555019	rs8853	0.0027	0.056
13q14.3	rs1638703	rs6561599	0.0067	0.19
18q11.2	rs9958656	rs17670370	0.0001	0.018
20q13.33	rs6061244	rs200383755	0.022	0.99

Correlation results are based on 8,700 whole-genome sequenced Icelanders.

Supplementary Table 4. Conditional analysis of eQTL results from the GTEx Portal, at BPH/LUTS loci where significant eQTL results are reported in the GTEx Portal.

Locus	Lead variant (Ea/Oa)	Strongest eQTL variant	Gene name	GTEx Tissue Sample	P-value	effect (beta)	P-value adjusted	effect adjusted
2p16.1	rs10180282 (T/C)	rs184838	AC007381.3	Skin_Sun_Exposed_Lower_leg	9.9E-05	-0.20	0.50	-0.050
5p15.33	rs381949 (A/G)	rs401681	TERT	Esophagus_Mucosa	2.1E-06	-0.29	0.27	0.16
5p15.33	rs381949 (A/G)	rs31490	CLPTM1L	Skin_Sun_Exposed_Lower_leg	2.7E-03	-0.096	0.72	-0.024
6p22.1	rs200476 (T/A)	rs1225618	ZNF603P	Muscle_Skeletal	7.1E-11	0.55	0.50	0.037
10q26.12	rs4548546 (T/C)	rs4751804	WDR11	Thyroid	5.3E-39	-0.71	0.86	0.018
11p15.5	rs72878024 (A/G)	rs1023430	BET1L	Esophagus_Muscularis	4.7E-04	-0.39	1.5E-03	-0.34
13q14.3	rs6561599 (C/G)	rs9535526	RNASEH2B	Muscle_Skeletal	2.6E-33	0.73	0.011	-0.22
20q13.33	rs6061244 (C/G)	rs6061555	GATA5	Esophagus_Muscularis	1.6E-04	0.32	0.22	0.13
20q13.33	rs6061244 (C/G)	rs13041686	GATA5	Skin_Sun_Exposed_Lower_leg	2.0E-03	0.26	0.20	0.071

Shown are eQTL association results (based on GTEx data) for lead variant before and after conditional analysis using as covariate the strongest eQTL variant at each loci.

Supplementary Table 5. Shown are association results for age at first TURP treatment for Icelandic men and variants genome-wide significantly associated with BPH/LUTS

Locus	Marker (EA/OA)	Position (B38)	Annotation / Nearby gene(s)	EAF	TURP Dx BPH only	
					Effect (years)	P-value
**a) Variants discovered in the unconditional GWAS analysis**						
2p16.1	rs2556378 (T/G)	60,535,367	Intron variant / *BCL11A*	0.149	-0.21	0.40
5p15.33	rs381949 (A/G)	1,322,353	Intron variant / *CLPTM1L*	0.421	0.36	0.053
5q22.1	rs10054105 (G/T)	111,573,636	Intergenic variant / *STARD4*	0.228	0.72	1.3E-03
5q31.1	rs677394 (G/C)	135,271,869	Intron variant / *C5orf66, H2AFY*	0.151	0.51	0.051
6p22.1	rs200476 (T/A)	27,800,570	Intergenic variant / *HIST1H2BL*	0.121	0.40	0.18
10p12.31	rs148678804 (A/G)	22,138,360	Intergenic variant / *DNAJC1*	0.039	-1.05	0.014
10q26.12	rs11199879 (C/T)	121,285,698	Intergenic variant / *FGFR2*	0.282	-0.32	0.11
11p15.5	rs72878024 (A/G)	199,492	Missense variant / *ODF3*	0.087	0.32	0.35
12q24.21	rs2555019 (T/C)	114,230,813	Intergenic variant / *TBX5*	0.455	-0.042	0.82
13q14.3	rs1638703 (C/G)	50,514,220	Intron variant / *DLEU1*	0.260	-0.058	0.77
17q12	rs11651052 (A/G)	37,742,390	Intron variant / *HNF1B*	0.464	-0.017	0.93
18q11.2	rs9958656 (T/C)	22,324,181	Intergenic variant / *GATA6*	0.444	-0.34	0.063
19q12	rs11084596 (C/T)	31,614,073	Intergenic variant / *THEG5*	0.330	-0.076	0.70
20q13.33	rs200383755 (C/G)	62,475,466	Missense variant / *GATA5*	0.012	2.67	0.013
**b) Variants discovered in the conditional GWAS analysis**						
2p16.1	rs10180282 (T/C)	60,351,708	Intergenic variant / *BCL11A*	0.466	-0.34	0.060
5p15.33	rs2853677 (G/A)	1,287,079	Intron variant / *TERT*	0.417	0.20	0.29
10p12.31	rs7906649 (G/A)	22,021,369	Intergenic variant / *EBLN1*	0.292	-0.51	9.7E-03
10q26.12	rs4548546 (T/C)	120,870,067	Intron variant / *WDR11*	0.287	-0.25	0.21
10q26.12	rs2981575 (G/A)	121,586,602	Intron variant / *FGFR2*	0.453	0.060	0.74
12q24.21	rs8853 (C/T)	114,671,102	3-prime UTR variant / *TBX3*	0.493	-0.27	0.14
13q14.3	rs6561599 (C/G)	50,904,782	Upstream gene variant / *RNASEH2B*	0.357	0.070	0.71
18q11.2	rs17670370 (G/T)	22,454,054	Intergenic variant / *CTAGE1*	0.280	-0.021	0.92
20q13.33	rs6061244 (C/G)	62,466,597	Intron variant / *GATA5*	0.363	0.29	0.12

The quantitative association result are based information about age at first TURP from 5,817 Icelandic men that have undergone TURP and have a histologically confirmed BPH (TURP Dx BPH). Shown are association results for the effect allele (EA) and its population frequency in Iceland (EAF).

Supplementary Table 6. GWAS and meta-analysis results for BPH/LUTS after removing men known to have been diagnosed with prostate cancer.

Locus	Marker (EA/OA)	Position (B38)	Annotation / Nearby gene(s)	EAF	P_{het} / I^2 (%)	Iceland		UK		Combined	
						OR (95% c.i.)	P-value	OR (95% c.i.)	P-value	OR (95% c.i.)	P-value
**a) Variants discovered in the unconditional GWAS analysis**											
2p16.1	rs2556378 (T/G)	60,535,367	Intron variant / *BCL11A*	0.154	0.58/0	1.11 (1.05, 1.17)	3.3E-04	1.13 (1.08, 1.17)	2.0E-09	1.12 (1.08, 1.16)	3.1E-12
5p15.33	rs381949 (A/G)	1,322,353	Intron variant / *CLPTM1L*	0.415	0.45/0	0.89 (0.86, 0.93)	3.1E-08	0.88 (0.85, 0.90)	2.3E-18	0.88 (0.86, 0.90)	5.7E-25
5q22.1	rs10054105 (G/T)	111,573,636	Intergenic variant / *STARD4*	0.213	0.59/0	0.91 (0.87, 0.96)	2.2E-04	0.9 (0.87, 0.93)	1.8E-08	0.9 (0.88, 0.93)	1.9E-11
5q31.1	rs677394 (G/C)	135,271,869	Intron variant / *C5orf66, H2AFY*	0.123	0.048/74	0.92 (0.87, 0.97)	3.5E-03	0.85 (0.81, 0.90)	1.3E-09	0.88 (0.85, 0.92)	1.3E-10
6p22.1	rs200476 (T/A)	27,800,570	Intergenic variant / *HIST1H2BL*	0.162	0.58/0	0.87 (0.81, 0.92)	6.0E-06	0.88 (0.85, 0.92)	1.2E-10	0.88 (0.85, 0.91)	4.1E-15
10p12.31	rs148678804 (A/G)	22,138,360	Intergenic variant / *DNAJC1*	0.035	0.031/79	1.21 (1.09, 1.33)	1.8E-04	1.38 (1.28, 1.49)	1.7E-16	1.31 (1.23, 1.39)	1.5E-18
10q26.12	rs11199879 (C/T)	121,285,698	Intergenic variant / *FGFR2*	0.252	0.0073/86	1.07 (1.03, 1.12)	1.4E-03	1.16 (1.12, 1.20)	2.5E-17	1.13 (1.10, 1.16)	5.5E-18
11p15.5	rs72878024 (A/G)	199,492	Missense variant / *ODF3*	0.080	0.33/0	0.83 (0.77, 0.89)	5.7E-07	0.87 (0.82, 0.92)	1.4E-06	0.85 (0.82, 0.89)	5.8E-12
12q24.21	rs2555019 (T/C)	114,230,813	Intergenic variant / *TBX5*	0.456	0.43/0	0.91 (0.87, 0.94)	1.8E-06	0.93 (0.90, 0.95)	2.6E-07	0.92 (0.90, 0.94)	3.0E-12
13q14.3	rs1638703 (C/G)	50,514,220	Intron variant / *DLEU1*	0.256	0.32/0	1.09 (1.04, 1.14)	1.5E-04	1.12 (1.08, 1.16)	1.2E-11	1.11 (1.08, 1.14)	1.3E-14
17q12	rs11651052 (A/G)	37,742,390	Intron variant / *HNF1B*	0.470	0.012/84	0.99 (0.95, 1.03)	5.2E-01	0.93 (0.90, 0.95)	3.4E-07	0.95 (0.93, 0.97)	7.5E-06
18q11.2	rs9958656 (T/C)	22,324,181	Intergenic variant / *GATA6*	0.430	0.37/0	1.09 (1.05, 1.14)	1.7E-05	1.12 (1.08, 1.15)	3.5E-13	1.11 (1.08, 1.13)	4.4E-17
19q12	rs11084596 (C/T)	31,614,073	Intergenic variant / *THEG5*	0.356	0.27/17	0.9 (0.86, 0.94)	4.8E-07	0.87 (0.84, 0.90)	7.2E-19	0.88 (0.86, 0.90)	3.6E-24
20q13.33	rs200383755 (C/G)	62,475,466	Missense variant / *GATA5*	0.0091	0.48/0	0.67 (0.55, 0.81)	3.1E-05	0.60 (0.48, 0.75)	6.0E-06	0.64 (0.55, 0.74)	1.0E-09
**b) Variants discovered in the conditional GWAS analysis**											
2p16.1	rs10180282 (T/C)	60,351,708	Intergenic variant / *BCL11A*	0.456	0.82/0	1.05 (1.01, 1.09)	1.5E-02	1.04 (1.01, 1.08)	4.4E-03	1.05 (1.02, 1.07)	1.8E-04
5p15.33	rs2853677 (G/A)	1,287,079	Intron variant / *TERT*	0.421	0.42/0	1.1 (1.06, 1.15)	1.8E-06	1.13 (1.09, 1.16)	3.0E-15	1.12 (1.09, 1.14)	4.0E-20
10p12.31	rs7906649 (G/A)	22,021,369	Intergenic variant / *EBLN1*	0.286	0.49/0	1.09 (1.04, 1.14)	1.6E-04	1.11 (1.07, 1.14)	5.5E-10	1.10 (1.07, 1.13)	4.8E-13
10q26.12	rs4548546 (T/C)	120,870,067	Intron variant / *WDR11*	0.310	0.053/73	1.07 (1.03, 1.12)	1.8E-03	1.13 (1.10, 1.16)	4.0E-15	1.11 (1.08, 1.14)	1.9E-16
10q26.12	rs2981575 (G/A)	121,586,602	Intron variant / *FGFR2*	0.427	0.64/0	0.95 (0.91, 0.99)	8.3E-03	0.94 (0.91, 0.97)	1.8E-05	0.94 (0.92, 0.96)	5.3E-07
12q24.21	rs8853 (C/T)	114,671,102	3-prime UTR variant / *TBX3*	0.494	0.97/0	1.07 (1.03, 1.12)	3.5E-04	1.08 (1.05, 1.11)	8.6E-07	1.08 (1.05, 1.10)	1.2E-09
13q14.3	rs6561599 (C/G)	50,904,782	Upstream gene variant / *RNASEH2B*	0.371	0.74/0	0.93 (0.89, 0.96)	2.1E-04	0.93 (0.91, 0.96)	7.2E-06	0.93 (0.91, 0.95)	6.1E-09
18q11.2	rs17670370 (G/T)	22,454,054	Intergenic variant / *CTAGE1*	0.262	0.15/51	1.04 (0.99, 1.08)	0.11	1.08 (1.04, 1.12	7.2E-06	1.06 (1.04,1.09)	5.4E-06
20q13.33	rs6061244 (C/G)	62,466,597	Intron variant / *GATA5*	0.386	0.74/0	0.93 (0.89, 0.97)	5.6E-04	0.92 (0.89, 0.95)	1.4E-07	0.92 (0.90, 0.95)	3.2E-10

Shown are results from a GWAS and meta-analysis for Iceland and the UK on men diagnosed with BPH/LUTS but not known to have been diagnosed with prostate cancer.
The sample sizes used are as follows: Iceland = 7,947 affected and 108,496 controls; UK = 10,191 affected and 171,717 controls; Combined = 18,138 affected and 277,213 controls

Supplementary Table 7. Association results BPH/LUTS and prostate cancer in combined study groups and for PSA-levels in Iceland only.

					BPH/LUTS			Prostate cancer			Serum levels of PSA Iceland only	
						Combined Iceland and UK			Combined Iceland and UK			
Locus	Marker (EA/OA)	Position (B38)	Annotation / Nearby gene(s)	EAF	P_{het} / I^2 (%)	OR (95% c.i.)	P-value	P_{het} / I^2 (%)	OR (95% c.i.)	P-value	Beta	P-value
**a) Variants discovered in the unconditional GWAS analysis**												
2p16.1	rs2556378 (T/G)	60,535,367	Intron variant / *BCL11A*	0.154	0.31/2	1.11 (1.08, 1.14)	3.3E-11	0.094/64	0.95 (0.92, 0.99)	0.026	0.044	4.6E-08*
5p15.33	rs381949 (A/G)	1,322,353	Intron variant / *CLPTM1L*	0.415	0.82/0	0.88 (0.86, 0.90)	2.3E-28	0.63/0	0.94 (0.91, 0.97)	6.7E-05	-0.038	4.5E-11*
5q22.1	rs10054105 (G/T)	111,573,636	Intergenic variant / *STARD4*	0.213	0.65/0	0.91 (0.88, 0.93)	3.5E-12	0.89/0	0.99 (0.96, 1.03)	0.61	-0.016	0.021
5q31.1	rs677394 (G/C)	135,271,869	Intron variant / *C5orf66, H2AFY*	0.123	0.034/78	0.88 (0.85, 0.92)	2.9E-11	0.17/47	0.98 (0.93, 1.02)	0.32	-0.020	0.013
6p22.1	rs200476 (T/A)	27,800,570	Intergenic variant / *HIST1H2BL*	0.162	0.23/30	0.88 (0.85, 0.90)	3.9E-17	0.3/6	0.92 (0.89, 0.96)	1.1E-04	-0.028	1.4E-03*
10p12.31	rs148678804 (A/G)	22,138,360	Intergenic variant / *DNAJC1*	0.035	0.16/50	1.34 (1.26, 1.41)	1.0E-22	0.71/0	1.04 (0.96, 1.13)	0.30	0.057	9.6E-05*
10q26.12	rs11199879 (C/T)	121,285,698	Intergenic variant / *FGFR2*	0.252	0.0092/85	1.13 (1.10, 1.16)	3.5E-20	0.48/0	0.98 (0.95, 1.01)	0.24	0.060	5.3E-21*
11p15.5	rs72878024 (A/G)	199,492	Missense variant / *ODF3*	0.080	0.20/40	0.85 (0.82, 0.89)	1.4E-12	0.40/0	0.92 (0.87, 0.97)	1.7E-03	-0.045	6.1E-06*
12q24.21	rs2555019 (T/C)	114,230,813	Intergenic variant / *TBX5*	0.456	0.93/0	0.93 (0.91, 0.95)	1.4E-10	0.38/0	1.05 (1.02, 1.08)	1.6E-03	0.015	8.3E-03
13q14.3	rs1638703 (C/G)	50,514,220	Intron variant / *DLEU1*	0.256	0.43/0	1.11 (1.08, 1.14)	2.0E-15	0.96/0	1.02 (0.99, 1.06)	0.23	0.024	1.8E-04*
17q12	rs11651052 (A/G)	37,742,390	Intron variant / *HNF1B*	0.470	0.24/29	0.93 (0.91, 0.95)	3.2E-10	0.87/0	0.82 (0.79, 0.84)	2.2E-40	-0.058	2.1E-24*
18q11.2	rs9958656 (T/C)	22,324,181	Intergenic variant / *GATA6*	0.430	0.97/0	1.11 (1.08, 1.13)	3.1E-18	0.61/0	1.00 (0.97, 1.03)	0.82	0.007	0.21
19q12	rs11084596 (C/T)	31,614,073	Intergenic variant / *THEG5*	0.356	0.34/0	0.88 (0.86, 0.90)	2.1E-24	0.77/0	1.00 (0.97, 1.03)	0.88	-0.041	1.4E-11*
20q13.33	rs200383755 (C/G)	62,475,466	Missense variant / *GATA5*	0.0091	0.77/0	0.62 (0.54, 0.71)	7.3E-12	0.69/0	0.91 (0.78, 1.07)	0.27	-0.084	8.4E-04*
**b) Variants discovered in the conditional GWAS analysis**												
2p16.1	rs10180282 (T/C)	60,351,708	Intergenic variant / *BCL11A*	0.456	0.55/0	1.05 (1.03, 1.08)	5.9E-06	0.46/0	1.01 (0.98, 1.04)	0.70	0.004	0.46
5p15.33	rs2853677 (G/A)	1,287,079	Intron variant / *TERT*	0.421	0.25/23	1.12 (1.09, 1.15)	1.5E-22	0.016/82.6	1.05 (1.02, 1.08)	1.8E-03	0.029	8.8E-07*
10p12.31	rs7906649 (G/A)	22,021,369	Intergenic variant / *EBLN1*	0.286	0.45/0	1.11 (1.08, 1.13)	1.5E-15	0.025/80.1	1.06 (1.02, 1.09)	1.2E-03	0.027	1.3E-05*
10q26.12	rs4548546 (T/C)	120,870,067	Intron variant / *WDR11*	0.310	0.11/62	1.1 (1.08, 1.13)	3.2E-15	0.47/0	1.04 (1.00, 1.07)	0.027	0.034	6.7E-08*
10q26.12	rs2981575 (G/A)	121,586,602	Intron variant / *FGFR2*	0.427	0.86/0	0.95 (0.92, 0.97)	2.7E-06	0.59/0	1.01 (0.98, 1.05)	0.46	-0.018	1.3E-03*
12q24.21	rs8853 (C/T)	114,671,102	3-prime UTR variant / *TBX3*	0.494	0.54/0	1.07 (1.05, 1.09)	3.6E-09	0.9/0	1.01 (0.98, 1.04)	0.41	0.024	2.9E-05*
13q14.3	rs6561599 (C/G)	50,904,782	Upstream gene variant / *RNASEH2B*	0.371	1.0/0	0.93 (0.91, 0.96)	1.4E-08	0.037/77	1.00 (0.97, 1.03)	0.86	0.0080	0.16
18q11.2	rs17670370 (G/T)	22,454,054	Intergenic variant / *CTAGE1*	0.262	0.37/0	1.06 (1.03,1.09)	5.3E-06	0.025/80	0.98 (0.93,1.03)	0.44	-0.009	0.15
20q13.33	rs6061244 (C/G)	62,466,597	Intron variant / *GATA5*	0.386	0.30/6	0.93 (0.91, 0.95)	8.1E-10	0.13/57	1.01 (0.98, 1.04)	0.39	-0.003	0.66

Combined Iceland and UK benign prostatic hyperplasia (BPH) study population consists of 20,621 affected and 280,541 controls. Combined Iceland and UK prostate cancer study population consists of 11,708 affected and 284,184 controls. Study population size of "Serum levels of prostate specific antigen (PSA) Iceland only" consists of 33,572 Icelandic males not known to have been diagnosed with symptomatic BPH/LUTS or prostate cancer**.**

*15 of the 23 BPH/LUTS variants reported here also associate with PSA levels at a Bonferroni corrected significance threshold ($P < 0.0022$)

Supplementary Table 8. Correlation information for BPH associated markers and markers reported for selected traits

Locus	BPH marker	Correlated marker	r^2	Reported trait	Reference
2p16.1	rs2556378	rs2556375	0.84	PSA levels	Hoffmann T, et al.[8].
5p15.33	rs381949	rs37004	0.38	PSA levels	Hoffmann T, et al.[8]
5p15.33	rs381949	rs401681	0.87	PSA levels	Gudmundsson J et al.[1]
5p15.33	rs381949	rs401681	0.87	MultiCancer risk	Rafnar Th, et al.[2]
5p15.33	rs381949	rs2736098	0.21	PSA levels	Gudmundsson J et al.[1]
5p15.33	rs381949	rs2736098	0.21	MultiCancer risk	Rafnar Th, et al.[2]
10p12.31	rs148678804	rs116940348	0.71	PSA levels	Hoffmann T, et al.[8]
10q26.12	rs11199879	rs10886902	0.99	PSA levels	Hoffmann T, et al.[8]
10q26.12	rs4548546	rs200367988	0.90	PSA levels	Hoffmann T, et al.[8]
12q24.21	rs2555019	rs1270884	0.81	PrCa risk	Eeles et al.[17]
12q24.21	rs2555019	rs10774740	0.51	AggrPrCa risk	Sonja I. Berndt et al.[18]
12q24.21	rs8853	rs11067228	0.64	PSA levels	Gudmundsson J et al.[1]/Hoffmann T, et al.[8]
13q14.3	rs1638703	rs202346	1.00	PSA levels	Hoffmann T, et al.[8]
17q12	rs11651052	rs4430796	0.91	PrCa risk	Gudmundsson J et al.[13]
17q12	rs11651052	rs4430796	0.91	PSA levels	Gudmundsson J et al.[1]
17q12	rs11651052	rs11263761	0.96	PSA levels	Hoffmann T, et al.[8]
19q12	rs11084596	rs11084596	1.00	PSA levels	Hoffmann T, et al.[8]

Shown are markers reported in main text (BPH markers) and correlated markers reported for the following traits: serum levels of prostate-specific antigen (PSA levels), cancer risk in multiple different organs (MultiCancer risk), risk of prostate cancer (PrCa risk), or aggressive prostate cancer (AggrPrCa risk)

Supplementary Table 9. Genetic correlation between phenotypes in Iceland and the UK

Iceland		UK		r_g	Standard error	P-value
**Phenotype**	**Count (n)**	**Phenotype**	**Count (n)**			
PSA-levels	33,572	BPH/LUTS	11,178	0.77	+/-0.12	2.6E-11
PSA-levels	33,572	PC	5,811	0.41	+/-0.10	6.1E-05
PC	5,897	BPH/LUTS	11,178	0.18	+/-0.14	0.18
BPH/LUTS	9,443	PC	5,811	0.17	+/-0.12	0.18
PC	5,897	PC	5,811	1.0	+/-0.18	1.5E-08
BPH/LUTS	9,443	BPH/LUTS	11,178	0.91	+/-0.16	9.5E-09

Shown is the genetic correlation (r_g) between different phenotypes in Iceland and the UK: benign prostatic hyperplasia/lower urinary tract symptoms (BPH/LUTS), serum levels of PSA (PSA-levels), prostate cancer (PC).

Supplementary Table 10. Results from testing the correlation between polygenic risk scores and a phenotype status.

PRSs	Phenotype	Effect (Beta)	OR (95% c.i.)	P-value
PC	BPH/LUTS	0.052	1.05 (1.018, 1.089)	0.0027
BPH/LUTS	PC	0.039	1.04 (0.999, 1.083)	0.059

Shown are results from testing the correlation between the polygenic risk score (PRS) for prostate cancer (PC) and the phenotype of benign prostatic hyperplasia/lower urinary tract symptoms (BPH/LUTS), and vice versa. The number of individuals belonging to each phenotype group is as follows: PC; including 3,464 patients and 43,029 controls, and BPH/LUTS; including 5,968 patients and 43,594 controls.

Supplementary References

1. Gudmundsson, J. *et al.* Genetic correction of PSA values using sequence variants associated with PSA levels. *Sci Transl Med* **2**, 62ra92 (2010).
2. Rafnar, T. *et al.* Sequence variants at the TERT-CLPTM1L locus associate with many cancer types. *Nat Genet* **41**, 221-7 (2009).
3. Figueroa, J.D. *et al.* Genome-wide association study identifies multiple loci associated with bladder cancer risk. *Hum Mol Genet* **23**, 1387-98 (2014).
4. Wang, Y. *et al.* Common 5p15.33 and 6p21.33 variants influence lung cancer risk. *Nat Genet* **40**, 1407-9 (2008).
5. Petersen, G.M. *et al.* A genome-wide association study identifies pancreatic cancer susceptibility loci on chromosomes 13q22.1, 1q32.1 and 5p15.33. *Nat Genet* **42**, 224-8 (2010).
6. Shiraishi, K. *et al.* A genome-wide association study identifies two new susceptibility loci for lung adenocarcinoma in the Japanese population. *Nat Genet* **44**, 900-3 (2012).
7. Wang, Z. *et al.* Imputation and subset-based association analysis across different cancer types identifies multiple independent risk loci in the TERT-CLPTM1L region on chromosome 5p15.33. *Hum Mol Genet* **23**, 6616-33 (2014).
8. Hoffmann, T.J. *et al.* Genome-wide association study of prostate-specific antigen levels identifies novel loci independent of prostate cancer. *Nat Commun* **8**, 14248 (2017).
9. Gaudet, M.M. *et al.* Common genetic variants and modification of penetrance of BRCA2-associated breast cancer. *PLoS Genet* **6**, e1001183 (2010).
10. Nam, R.K. *et al.* New variants at 10q26 and 15q21 are associated with aggressive prostate cancer in a genome-wide association study from a prostate biopsy screening cohort. *Cancer Biol Ther* **12**, 997-1004 (2011).
11. Bamshad, M. *et al.* Mutations in human TBX3 alter limb, apocrine and genital development in ulnar-mammary syndrome. *Nat Genet* **16**, 311-5 (1997).
12. Feng, S. & Cao, Z. Is the role of human RNase H2 restricted to its enzyme activity? *Prog Biophys Mol Biol* **121**, 66-73 (2016).
13. Gudmundsson, J. *et al.* Two variants on chromosome 17 confer prostate cancer risk, and the one in TCF2 protects against type 2 diabetes. *Nat Genet* **39**, 977-83 (2007).
14. Wei, D. *et al.* GATA5 loss-of-function mutations underlie tetralogy of fallot. *Int J Med Sci* **10**, 34-42 (2013).
15. International HapMap, C. *et al.* A second generation human haplotype map of over 3.1 million SNPs. *Nature* **449**, 851-61 (2007).
16. Pruim, R.J. *et al.* LocusZoom: regional visualization of genome-wide association scan results. *Bioinformatics* **26**, 2336-7 (2010).
17. Eeles, R.A. *et al.* Identification of 23 new prostate cancer susceptibility loci using the iCOGS custom genotyping array. *Nat Genet* **45**, 385-91, 391e1-2 (2013).
18. Berndt, S.I. *et al.* Two susceptibility loci identified for prostate cancer aggressiveness. *Nat Commun* **6**, 6889 (2015).
