# Supplementary material for: Genome-wide associations for benign prostatic hyperplasia reveal a genetic correlation with serum levels of PSA
Source: Nat Commun. 2018 Nov 8;9:4568. doi: 10.1038/s41467-018-06920-9 (PMC6224563; doi:10.1038/s41467-018-06920-9)
Supplement: Supplementary file 2 — Description of Additional Supplementary Files [file 41467_2018_6920_MOESM2_ESM.docx]

**Description of Additional Supplementary Files**

File Name: Supplementary Data 1

Description: A table with functional annotation of lead- and correlated variants at BPH/LUTS risk loci.

File Name: Supplementary Data 2

Description: A summary table with results for BPH/LUTS risk variants and H3K27-acetylated regions, Dnase hypersensitivity sites and topologically associating domains in prostate derived cells/cell-lines.
